# Identification of 2-(thiophen-2-yl)acetic Acid-Based Lead Compound for mPGES-1 Inhibition

**DOI:** 10.3389/fchem.2021.676631

**Published:** 2021-05-07

**Authors:** Simone Di Micco, Stefania Terracciano, Dafne Ruggiero, Marianna Potenza, Maria C. Vaccaro, Katrin Fischer, Oliver Werz, Ines Bruno, Giuseppe Bifulco

**Affiliations:** ^1^European Biomedical Research Institute of Salerno (EBRIS), Salerno, Italy; ^2^Dipartimento di Farmacia, University degli Studi di Salerno, Fisciano, Italy; ^3^Institute of Pharmacy, Friedrich-Schiller-University Jena, Jena, Germany

**Keywords:** fragment-based approach, Suzuki-Miyaura cross-coupling, mPGES-1 inhibitors, anti-inflammatory drugs, anticancer agents

## Abstract

We report the implementation of our *in silico*/synthesis pipeline by targeting the glutathione-dependent enzyme mPGES-1, a valuable macromolecular target in both cancer therapy and inflammation therapy. Specifically, by using a virtual fragment screening approach of aromatic bromides, straightforwardly modifiable by the Suzuki-Miyaura reaction, we identified 3-phenylpropanoic acid and 2-(thiophen-2-yl)acetic acid to be suitable chemical platforms to develop tighter mPGES-1 inhibitors. Among these, compounds **1c** and **2c** showed selective inhibitory activity against mPGES-1 in the low micromolar range in accordance with molecular modeling calculations. Moreover, **1c** and **2c** exhibited interesting IC_50_ values on A549 cell lines compared to CAY10526, selected as reference compound. The most promising compound **2c** induced the cycle arrest in the G_0_/G_1_ phase at 24 h of exposure, whereas at 48 and 72 h, it caused an increase of subG_0_/G_1_ fraction, suggesting an apoptosis/necrosis effect.

## Introduction

**Figure 1:**
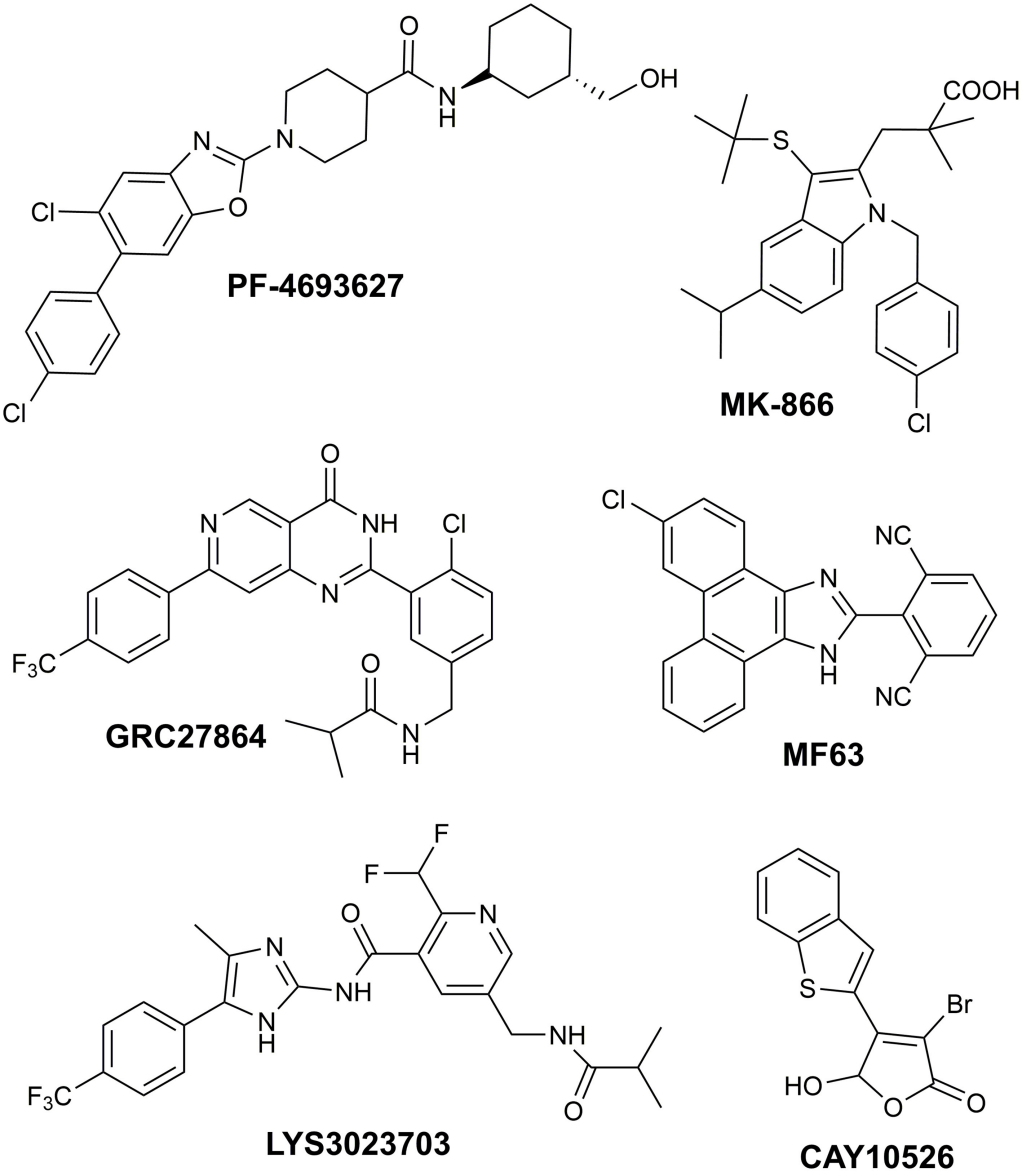
Molecular structure of known mPGES-1 inhibitors.

Recently, we reported the successful identification of new mPGES-1 (Microsomal prostaglandin E synthase-1) inhibitors endowed with 1-fluoro-2,4-dinitro-biphenyl scaffold (Di Micco et al., 2018), acting in the low micromolar range in cell-free assays. Our hit discovery strategy was based on the virtual screening of commercially available fragments featuring aryl halide moieties, which represent the basic partners for the Suzuki-Miyaura reactions (Miyaura and Suzuki, 1995), a very suitable synthetic strategy leading to platforms highly prone to further chemical modifications (Giordano et al., 2018b). The obtained encouraging results prompted us to expand the compounds collection aiming to improve the affinity toward the biological target to find new potential drug candidates. mPGES-1 is an inducible transmembrane enzyme, belonging to the MAPEG [membrane-associated proteins in eicosanoid and glutathione (GSH) metabolism] protein family (Jakobsson et al., 2000). This glutathione-dependent enzyme catalyzes the last step of the biosynthesis of prostaglandin E_2_ (PGE_2_), resulting particularly upregulated in inflammation (Gerstmeier et al., 2019), in tumors (Nakanishi et al., 2010), and other pathologies such as Alzheimer's disease, arthritis, burn injury, atherosclerosis (Akitake et al., 2013). Besides the involvement of mPGES-1 in inflammation-related disorders, its biosynthetic product, PGE_2_, has been demonstrated to affect cancer cell proliferation and tumor development in several animal models. In recent years, mPGES-1 has been gaining great attention as a strategic therapeutic target, being the inducible terminal synthase in the production of PGE_2_. Indeed, its inhibition selectively affects the PGE_2_ upregulated by pathological conditions, while keeping the tissue levels of other important prostanoid molecules (Trebino et al., 2003), which are responsible for key cellular physiological functions. This special feature allows circumventing the typical gastrointestinal and cardiovascular side effects of COX-1/2 and selective COX-2 inhibitors (Di Micco et al., 2016). Despite the great interest in this biological target and the many efforts focused on the development of potential mPGES-1 binders (Figure 1), so far, no mPGES-1 inhibitors for clinical use have reached the market (Bergqvista et al., 2020). MK-886 is the first synthetic mPGES-1 inhibitor structurally based on an indole-carboxylic acid scaffold (Mancini et al., 2001), which led to some phenanthrene imidazole derivatives such as MF63 (Figure 1; Psarra et al., 2017), but no clinical data are available. Among the benzoxazole piperidine carboxamides, PF4693627 (Figure 1) showed good pharmacodynamic and pharmacokinetic properties, and it has been proposed for use in clinical studies for the treatment of rheumatoid arthritis and osteoarthritis (Arhancet et al., 2013). Some of the proposed inhibitors reached Phase 1, such as the imidazole derivative LY3023703 and the substituted pyrimidine GRC 27864 (Figure 1; Chini et al., 2020). Overall, the structural differences in mouse and human mPGES-1 hamper the development of candidates, both working in preclinical and clinical stages (Bergqvista et al., 2020). Indeed, different research groups from academia and pharma companies have introduced in their drug discovery pipeline biological assays on mouse/rat cell lines (Kim et al., 2016; Ding et al., 2018; Larsson et al., 2019) and *in vivo* experiments on guinea pig or human mPGES-1 knock-in mice (Xu et al., 2008; Bergqvista et al., 2020). Furthermore, the high plasma protein binding of lead structures also represents a big deal in drug advancement (Koeberle et al., 2016; Di Francesco et al., 2020). Thus, the discovery of new promising chemotypes, hopefully endowed with cross-species activity, for clinical practice is highly requested. In this scenario, many efforts have been made by our research group in order to gain insights into the structural requirements needed to assure an optimal interaction between a ligand and the biological target under investigation (Di Micco et al., 2016, 2018; Gerstmeier et al., 2019; Chini et al., 2020). Different chemical entities, with a promising anti-MPGES-1 activity, have been identified and are currently under further investigation to deepen their biological and pharmacological profile.

However, among the several drug discovery strategies available to perform this structure–activity relationship task, one approach is to expand the chemical diversity of the potential ligands, by developing several compound collections to gather as much information as possible. Thereby, following our fragment-based approach, we succeeded in identifying two very promising novel chemotypes whose antiproliferative effect is strongly related to their ability to inhibit mPGES-1, which was not demonstrated in the precedent contribution (Di Micco et al., 2018). Moreover, we implemented our *in silico* workflow by widening the input aryl bromides and inserting further filters to identify new potential molecular seeds to build the lead compounds. The implemented approach allowed for the wide exploration of the chemical diversity, resulting in the identification of two starting fragments that are structurally different, unlike the previous work (Di Micco et al., 2018).

## Materials and Methods

### Computational Details

The 3D structures of **1a-f** and **2a**-**f** were built by means of Build Panel of Maestro (version 11) and refined by applying the following: the OPLS3 force field (Harder et al., 2016), Polak–Ribier conjugate gradient algorithm (maximum derivative <0.001 kcal/mol), and the GB/SA (generalized Born/surface area) (Still et al., 1990) solvent treatment for the presence of H_2_O. Then, **1a-f** and **2a**-**f** were processed with LigPrep (2017), generating all possible tautomers, stereoisomers, and protonation states at a pH of 7.0 ± 1.0. As for the protein model, the x-ray structure of mPGES-1 (PDB ID: 4AL0) was employed and processed by means of Protein Preparation Wizard (2017) and Sastry et al. (2013): missing side chains and loops were checked; bond order was assigned and all hydrogens were added; and residue alternate positions were checked considering the A conformation. The charges of the side chains were attributed according to their pK_a_ at pH 7.0 and the hydrogen bond network was refined through the optimize option. All water molecules were deleted. Glide (2017) and Friesner et al. (2006) was used for molecular docking calculations. The docking methodology was previously validated (Di Micco et al., 2018) by docking two co-crystallized low-size molecules (Giordano et al., 2018a; Di Micco et al., 2019) endowed with a molecular size similar to that of screened fragments. We sized the inner and outer receptor grid boxes to be 10 and 17 Å, respectively, and centered between chain A and C on the x-, y-, and z-coordinates: 3.04, 21.16, −7.75, respectively. For each step of our workflow, firstly we used Standard Precision (SP), applying default parameters and generating one pose per ligand. Secondly, the generated poses from the SP calculations were employed as input conformations for two rounds of predictions in the Extra Precision (XP) Glide mode, considering the halogen atoms to be the acceptor and donor of bonds. The ligands were treated as flexible, allowing the sole trans conformation for amide bonds, also sampling the nitrogen inversion and ring conformations (with an energy cutoff of 2.5 kcal/mol). The enhanced sampling mode was applied, maintaining 10,000 poses/ligand for the initial step of docking and selecting 1,000 poses per ligand for energy minimization. For each ligand, 1,000 maximum output structures were kept by using 0.15 as the partial charge cutoff and 0.8 as the scaling factor for van der Waals radii. Post-docking optimization was performed on docked conformations, taking into account 100 maximum number of poses and applying 0.5 kcal/mol as a cutoff for rejecting obtained minimized poses. The following energy contributions were accounted for: aromatic H and halogen bonds (as donor and acceptor); the reward of intramolecular H-bonds; and Epik state penalty. Maestro (version 11) was employed for docking outcome analysis and for the generation of figures.

### Chemistry

All commercially available starting materials were purchased from Sigma-Aldrich and used as received. All solvents used for the synthesis were of HPLC grade and purchased from Sigma-Aldrich. The NMR spectra (^1^H, ^13^C) were recorded using the Bruker Avance 400, 500, 600 MHz instruments (Billerica, MA, United States), *T* = 298 K. The compounds were dissolved in 0.5 mL of DMSO-d_6_, CD_3_OD, CDCl_3_ (Sigma-Aldrich, 99.8 Atom %D). Coupling constants (*J*) are reported in Hertz, and the chemical shifts are expressed in ppm on the delta (δ) scale relative to the solvent peak as the internal reference. Multiplicities are reported as follows: s, singlet; d, doublet; t, triplet; m, multiplet; dd, doublet of doublet; ddd, doublet of doublet of doublet. Mass spectrometry experiments were performed using an HPLC–MS system Q-ToF Premiere instrument (Waters, Co., Milford, MA, United States) equipped with an ESI source and Waters pump system. The reactions were monitored on silica gel 60 F254 plates (Merck, Darmstadt, Germany) and the spots were visualized under UV light (λ = 254, 365 nm). Analytical and semi-preparative reversed-phase HPLC was performed on Agilent Technologies 1200 Series high-performance liquid chromatography using a Nucleodur, C8 reversed-phase column (100 mm × 2 mm, 4 μM, 80 Å, flow rate = 1 mL/min; 250 mm × 10 mm, 4 μM, 80 Å, flow rate = 4 mL/min, respectively, Phenomenex®). The binary solvent system (A/B) was as follows: 0.1% TFA in water (A) and 0.1% TFA in CH_3_CN (B). The absorbance was detected at 220/240 nm. The purity of all the tested compounds (>98%) was determined by HPLC analysis.

### General Procedure for the Suzuki-Miyaura Reaction

First, 3-(2-bromophenyl)propanoic acid (**1**) or 2-(4-bromothiophen-2-yl)acetic acid (**2**) (1.0 equiv.), the appropriate substituted boronic acid (**a-f**) (1.2 equiv.), K_2_CO_3_ (2 equiv.), and Pd(PPh_3_)_4_ (0.05 equiv.) were placed in a 25 mL round-bottom flask equipped with a stir bar in the air. The flask was evacuated and refilled with N_2_ five times. A mixture of dioxane/H_2_O (3.0 and 1.5 mL; rate 2:1) was placed in another 25 mL round-bottom flask, which was also evacuated and refilled with N_2_ five times. Finally, the dioxane/H_2_O mixture was added using a syringe in the flask with the powders. The reaction was fitted with a condenser, immersed in an oil bath, and refluxed overnight. The reaction mixture was cooled, diluted with acidified water (20 mL), and filtered. The filtrate was washed with CH_3_OH and CHCl_3_ (20 mL), dried over anhydrous Na_2_SO_4_, and concentrated under reduced pressure. The desired compounds **1a**-**1f** and **2a**-**2f** were confirmed by analytical RP-HPLC (Nucleodur, C8 reversed-phase column: 100 mm × 2 mm, 4 μM, 80 Å, flow rate = 1 mL/min). HPLC purification was performed by semi-preparative reversed-phase HPLC (Nucleodur, C8 reversed-phase column: 250 mm × 10 mm, 4 μM, 80 Å, flow rate = 4 mL/min). The final products were obtained with high purity (>98%) as detected by HPLC analysis and were fully characterized by electrospray ionization mass spectrometry (ESI-MS) and NMR spectra.

### 3-(4′-(4-methylphenylsulfonamido)-[1,1′-biphenyl]-2-yl)propanoic Acid (1a)

Compound **1a** was obtained by following the general procedure as a brown solid (30% yield after HPLC). RP-HPLC t_R_ = 31.8 min, gradient condition: from 5% B to 100% B in 60 min, flow rate of 4 mL/min, λ = 240 nm. ^1^H NMR (400 MHz, CD_3_OD): δ = 7.55 (d, *J* = 8.2 Hz, 2H), 7.20 (d, *J* = 8.0 Hz, 2H), 7.16–7.13 (m, 2H), 7.10 (ddd, *J* = 8.8, 5.3, 3.7 Hz, 1H), δ 7.06–7.01 (m, 4H), 7.00–6.97 (m, 1H), 2.74 (t, *J* = 7.9 Hz, 2H), 2.26 (s, 3H), 2.24 (t, *J* = 7.9 Hz, 2H). ^13^C NMR (100 MHz, CD_3_OD): δ = 173.5, 143.6 (2C), 141.1, 138.0, 137.7, 136.7, 129.7, 129.4 (2C), 129.2 (2C), 128.8, 127.3, 126.85 (2C), 126.0, 120.9 (2C), 34.5, 28.0, 20.0. ESI-MS: calculated for C_22_H_21_NO_4_S 395.47; found m/z = 394.93 [M-H]^−^.

### 3-(3′-(4-methylphenylsulfonamido)-[1,1′-biphenyl]-2-yl)propanoic Acid (1b)

Compound **1b** was obtained by following the general procedure as a dark brown solid (60% yield after HPLC). RP-HPLC t_R_ = 35.3 min, gradient condition: from 5% B to 100% B in 60 min, flow rate of 4 mL/min, λ = 240 nm. ^1^H NMR (400 MHz, CD_3_OD): δ = 7.67–7.64 (m, 2H), 7.32–7.20 (m, 6H), 7.10–6.99 (m, 4H), 2.80 (t, *J* = 7.9 Hz, 2H), 2.39 (s, 3H), 2.32 (t, *J* = 7.9 Hz, 2H). ^13^C NMR (100 MHz, CD_3_OD): δ = 173.4, 143.6, 142.5, 141.2, 137.6, 137.5, 136.6, 129.5, 129.2 (2C), 128.9, 128.7, 127.5, 126.9 (2C), 126.0, 125.2, 121.9, 119.9, 34.6, 27.9, 20.0. ESI-MS: calculated for C_22_H_21_NO_4_S 395.47; found m/z = 394.12 [M-H]^−^.

### 3-(3′-((2-chlorobenzyl)oxy)-[1,1′-biphenyl]-2-yl)propanoic Acid (1c)

Compound **1c** was obtained by following the general procedure as a yellow solid (65% after HPLC). RP-HPLC t_R_ = 40.9 min, gradient condition: from 5% B to 100% B in 60 min, Flow rate of 4 mL/min, λ = 240 nm. ^1^H NMR (400 MHz, CD_3_OD): δ = 7.60–7.55 (m, 1H), 7.48–7.39 (m, 1H), δ 7.37–7.26 (m, 5H), 7.23 (t, *J* = 4.6 Hz, 1H), 7.17 (d, *J* = 7.1 Hz, 1H), 7.04–6.97 (m, 1H), 6.91 (d, *J* = 7.1 Hz, 2H), 5.21 (s, 2H), 2.88 (t, *J* = 7.9 Hz, 2H), 2,38 (t, *J* = 7.9 Hz, 2H). ^13^C NMR (125 MHz, CD_3_OD): δ = 175.4, 158.4, 143.1, 141.6, 137.9, 134.7, 132.6, 129.6, 129.1, 129.1 (2C), 129.0, 128.8, 127.3, 126.8, 125.8, 121.8, 115.4, 113.3, 66.9, 34.9, 28.1. ESI-MS: calculated for C_22_H_19_ClO_3_ 366.84; found m/z = 367.09 [M+H]^+^.

### 3-(3′-benzoyl-[1,1′-biphenyl]-2-yl)propanoic Acid (1d)

Compound **1d** was obtained by following the general procedure as a dark brown solid (45% after HPLC). RP-HPLC t_R_ = 36.5 min, gradient condition: from 5% B to 100% B in 60 min, flow rate of 4 mL/min, λ = 240 nm. ^1^H NMR (400 MHz, CD_3_OD): δ = 7.91–7.80 (m, 4H), 7.68 (dd, *J* = 8.4, 6.3 Hz, 1H), 7.57 (t, *J* = 7.7, 2H), 7.51 (dd, *J* = 8.3, 3.1 Hz, 2H), 7.41–7.33 (m, 3H), 7.24 (d, *J* = 7.2 Hz, 1H), 2.96 (t, *J* = 7.9 Hz, 2H), 2.45 (t, *J* = 7.9 Hz, 2H). ^13^C NMR (150 MHz, CD_3_OD): δ = 196.9, 175.0, 146.3, 140.9, 137.8, 137.5, 136.0, 132.4, 129.7 (2C), 129.6 (2C), 129.5, 129.0 (2C), 128.9, 128.1 (2C), 127.9, 126.1, 34.7, 28.0. ESI-MS: calculated for C_22_H_18_O_3_ 330.38; found m/z = 331.09 [M+H]^+^.

### 3-(4′-(phenoxymethyl)-[1,1′-biphenyl]-2-yl)propanoic Acid (1e)

Compound **1e** was obtained by following the general procedure as a red solid (65% after HPLC). RP-HPLC t_R_ = 39.7 min, gradient condition: from 5% B to 100% B in 60 min, flow rate of 4 mL/min, λ = 240 nm. ^1^H NMR (400 MHz, CD_3_OD): δ = 7.43–7.37 (m, 2H), 7.23–7.11 (m, 7H), 7.05 (d, *J* = 7.4 Hz, 1H), 6.91 (d, *J* = 8.0 Hz, 2H), 6.83 (t, *J* = 7.4 Hz, 1H), 4.75 (s, 2H), 2.79 (t, *J* = 8.0 Hz, 2H), 2.28 (t, *J* = 8.0 Hz, 2H). ^13^C NMR (100 MHz, CD_3_OD): δ = 173.5, 158.8, 141.6, 141.2, 137.7, 136.2, 129.8, 129.1 (2C), 128.9 (2C), 128.8, 127.3, 127.1 (2C), 126.0, 120.6, 114.6 (2C), 69.3, 34.5, 28.0. ESI-MS: calculated for C_22_H_20_O_3_; found 332.39 m/z = 372.08 [M+K]^+^.

### 3-(3′-(benzyloxy)-[1,1′-biphenyl]-2-yl)propanoic Acid (1f)

Compound **1f** was obtained by following the general procedure as a dark green solid (60% after HPLC). RP-HPLC t_R_ = 39.3 min, gradient condition: from 5% B to 100% B in 60 min, flow rate of 4 mL/min, λ = 240 nm. ^1^H NMR (400 MHz, CD_3_OD): δ = 7.33 (d, *J* = 7.5 Hz, 2H), 7.25 (t, *J* = 7.4 Hz, 2H), 7.22–7.16 (m, 4H), 7.15–7.09 (m, 1H), 7.04 (d, *J* = 7.6 Hz, 1H), 6.89 (d, *J* = 8.5 Hz, 1H), 6.81–6.74 (m, 2H), 4.75 (s, 2H), 2.75 (t, *J* = 8.0 Hz, 2H), 2.25 (t, *J* = 8.0 Hz, 2H). ^13^C NMR (100 MHz, CD_3_OD): δ = 175.4, 158.6, 143.0, 141.7, 137.8, 137.3, 129.6, 128.9, 128.7, 128.1 (2C), 127.4, 127.2, 127.1 (2C), 126.0, 121.5, 115.4, 113.4, 69.6, 34.9, 28.1. ESI-MS: calculated for C_22_H_20_O_3_; found 332.39 m/z = 333.17 [M+H]^+^.

### 2-(4-(4-(4-methylphenylsulfonamido)phenyl)thiophen-2-yl)acetic Acid (2a)

Compound **2a** was obtained by following the general procedure as a brown solid (55% after HPLC). RP-HPLC t_R_ = 39.3 min, gradient condition: from 5% B to 100% B in 60 min, flow rate of 4 mL/min, λ = 240 nm. ^1^H NMR (400 MHz, CDCl_3_): δ = 7.67 (d, *J* = 7.9 Hz, 2H), 7.45 (d, *J* = 8.0 Hz, 2H), 7.27–7.21 (m, 3H), 7.09 (d, *J* = 8.1 Hz, 2H), 6.63 (s, 1H), 3.92 (s, 2H), 2.40 (s, 3H). ^13^C NMR (100 MHz, CD_3_OD): δ = 172.7, 143.6, 140.8, 136.8, 136.7, 136.5, 132.4, 129.1 (2C), 126.9 (2C), 126.3 (2C), 125.5, 121.3 (2C), 119.00, 34.7, 20.0. ESI-MS: calculated for C_19_H_17_NO_4_S_2_; found 387.47 m/z = 388.10 [M+H]^+^.

### 2-(4-(3-(4-methylphenylsulfonamido)phenyl)thiophen-2-yl)acetic Acid (2b)

Compound **2b** was obtained by following the general procedure as a dark brown solid (40% after HPLC). RP-HPLC t_R_ = 26.9 min, gradient condition: from 5% B to 100% B in 60 min, flow rate of 4 mL/min, λ = 240 nm. ^1^H NMR (400 MHz, CDCl_3_): δ = 7.59 (d, *J* = 8.0 Hz, 2H), 7.25–7.12 (m, 5H), 7.10 (s, 1H), 6.89 (d, *J* = 7.8 Hz, 1H), 6.55 (s, 1H), 3.83 (s, 2H), 2.30 (s, 3H). ^13^C NMR (100 MHz, CD_3_OD): δ = 172.7, 143.7, 140.9, 138.1, 137.0, 136.8, 136.6, 129.2 (2C), 129.1, 126.9 (2C), 125.5, 122.0, 119.7, 119.3, 118.4, 34.6, 20.0. ESI-MS: calculated for C_19_H_17_NO_4_S_2_; found 387.47 m/z = 388.08 [M+H]^+^.

### 2-(4-(3-((2-chlorobenzyl)oxy)phenyl)thiophen-2-yl)acetic Acid (2c)

Compound **2c** was obtained by following the general procedure as a dark brown solid (45% after HPLC). RP-HPLC t_R_ = 27.9 min, gradient condition: from 5% B to 100% B in 60 min, flow rate of 4 mL/min, λ = 240 nm. ^1^H NMR (400 MHz, DMSO-d_6_): δ = 7.76 (d, *J* = 1.5 Hz, 1H), 7.66–7.62 (m, 1H), 7.55–7.51 (m, 1H), 7.42–7.39 (m, 3H), 7.36–7.31 (m, 2H), 7.30–7.26 (m, 1H), 6.95 (dd, *J* = 8.0, 2.5 Hz, 1H), 5.22 (s, 2H), 3.85 (s, 2H). ^13^C NMR (150 MHz, CD_3_OD): δ = 171.0, 157.9, 145.1, 136.5, 135.2, 134.8, 132.6, 129.0, 128.9, 128.6, 128.3, 128.1, 126.7, 126.4, 125.8, 116.6, 115.9, 66.7, 39.0. ESI-MS: calculated for C_19_H_15_ClO_3_S; found 358.84 m/z = 359.29 [M+H]^−^.

### 2-(4-(4-benzoylphenyl)thiophen-2-yl)acetic Acid (2d)

Compound **2d** was obtained by following the general procedure as a dark brown solid (40% after HPLC). RP-HPLC t_R_ = 35.3 min, gradient condition: from 5% B to 100% B in 60 min, flow rate of 4 mL/min, λ = 240 nm. ^1^H NMR (400 MHz, CD_3_OD): δ = 7.85–7.78 (m, 6H), 7.74–7.72 (m, 1H), 7.67 (t, *J* = 7.5 Hz, 1H), 7.56 (t, *J* = 7.6 Hz, 2H), 7.43 (s, 1H), 3.96 (s, 2H). ^13^C NMR (100 MHz, CD_3_OD): δ = 196.6, 171.2, 140.4, 139.9, 137.7, 136.9, 135.6, 132.3, 130.5 (2C), 129.5 (2C), 128.1 (2C), 125.7, 125.6 (2C), 121.7, 34.5. ESI-MS: calculated for C_19_H_14_O_3_S; found 322.38 m/z = 321.07 [M-H]^+^.

### 2-(4-(4-(phenoxymethyl)phenyl)thiophen-2-yl)acetic Acid (2e)

Compound **2e** was obtained by following the general procedure as a dark brown solid (55% after HPLC). RP-HPLC t_R_ = 37.8 min, gradient condition: from 5% B to 100% B in 60 min, flow rate of 4 mL/min, λ = 240 nm. ^1^H NMR (400 MHz, CD_3_OD): δ = 7.56–7.52 (m, 2H), 7.40 (s, 1H), 7.36 (d, *J* = 7.7 Hz, 2H), 7.22 (s, 1H), 7.17 (t, *J* = 8.0 Hz, 2H), 6.89 (d, *J* = 8.0 Hz, 2H), 6.83 (t, *J* = 7.7 Hz, 1H), 4.97 (s, 2H), 3.76 (s, 2H). ^13^C NMR (100 MHz, CD_3_OD): δ = 179.3, 160.0, 145.5, 141.9, 141.9, 137.7, 129.6 (2C), 128.8 (2C), 128.2, 128.8 (2C), 127.7, 121.7, 115.8 (2C), 70.4, 40.3. ESI-MS: calculated for C_19_H_16_O_3_S; found 324.39 m/z = 325.08 [M-H]^−^.

### 2-(4-(3-(benzyloxy)phenyl)thiophen-2-yl)acetic Acid (2f)

Compound **2f** was obtained by following the general procedure as a dark brown solid (40% after HPLC). RP-HPLC t_R_ = 36.3 min, gradient condition: from 5% B to 100% B in 60 min, flow rate of 4 mL/min, λ = 240 nm. ^1^H NMR (400 MHz, CDCl_3_): δ = 7.38 (d, *J* = 7.0 Hz, 2H), 7.32 (t, *J* = 7.4 Hz, 2H), 7.28–7.24 (m, 2H), 7.21–7.16 (m, 2H), 7.11–7.07 (m, 2H), 6.83 (dd, *J* = 8.3, 2.5 Hz, 1H), 5.03 (s, 2H), 3.84 (s, 2H). ^13^C NMR (150 MHz, CD_3_OD): δ = 172.9, 159.2, 141.5, 137.4, 137.2, 136.7, 129.4, 128.1 (2C), 127.4, 127.2 (2C), 125.7, 119.5, 118.5, 113.1, 112.4, 69.6, 34.4. ESI-MS: calculated for C_19_H_16_O_3_S; found 324.39 m/z = 325.20 [M-H]^−^.

### Biological Assay for mPGES-1 Activity

mPGES-1 was obtained by differential centrifugation from interleukin (IL)-1β-stimulated A549 cell microsomes (Koeberle et al., 2008). Firstly, the A549 cells were stimulated by IL-1β (1 ng/mL) for 48 h; then, the cells were harvested and sonicated. The obtained homogenate underwent differential centrifugation at 10,000 × g for 10 min, whereas the supernatant was centrifuged at 174,000 × g for 1 h at 4°C. The microsomal fraction (pellet) was resuspended in 1 mL of homogenization buffer (0.1 M potassium phosphate buffer, pH 7.4, 60 μg/mL soybean trypsin inhibitor, 1 mM phenylmethanesulfonyl fluoride, 250 mM sucrose, 2.5 mM glutathione, 1 μg/mL leupeptin), and the total protein concentration was assessed. The microsomes were resuspended in potassium phosphate buffer (0.1 M, pH 7.4) containing 2.5 mM of glutathione. Compounds **1a**-**1f** and **2a**-**2f** or dimethyl sulfoxide (DMSO; as vehicle control) were added, and 15 min later, the reaction (4°C, 100 μL total volume) started following the addition of 20 μM of PGH_2_. After 1 min, 100 μL of stop solution (40 mM FeCl_2_, 80 mM citric acid, and 10 μM 11β-PGE2) were dropped in at 4°C. PGE_2_ was firstly separated by solid-phase extraction and then analyzed by RP-HPLC as previously described (Koeberle et al., 2008).

### Cell Line

Human lung carcinoma A549 cell lines were purchased from Cell Application Inc., Sigma-Aldrich, and Merck (Darmstadt, Germany) and maintained in Dulbecco's Modified Eagle Medium (DMEM) supplemented with 10% heat-inactivated fetal bovine serum (FBS; Invitrogen, Carlsbad, CA, United States) in a 5% CO_2_ humid atmosphere. To ensure logarithmic growth, the cells were subcultured every 2 days. The cell line was tested for mycoplasma using PCR analysis.

### Inhibition of PGE_2_ Production in Cells

The A549 cells were plated at 10,000 cells/well in DMEM with 10% FBS medium in a 96-well plate. After overnight incubation, the cells were treated with 10 μM of compounds **1c** and **2c** in DMEM 1% FBS and IL-1β (10 ng/mL) to upregulate the expression of PG synthases.

After 24 h, the amount of PGE_2_ released in the supernatant was evaluated by using a commercially available enzyme immunoassay kit (Prostaglandin E_2_ EIA kit Monoclonal, Cayman Chemical Company, Ann Arbor, MI, United States).

### Activity Assays of Isolated COX-1 and COX-2

Compounds **1c** and **2c** were submitted for the evaluation of their activity on cyclooxygenases (COXs), specifically using isolated ovine COX-1 and recombinant human COX-2. The COXs were diluted to a final concentration of 50 U/mL for COX-1 and 20 U/mL for COX-2 in Tris buffer (100 mM, pH = 8) supplemented with glutathione (5 mM), EDTA (100 μM) and hemoglobin (5 μM) and pre-incubated with vehicle (0.1% DMSO) or test compounds (at 10 μM) over 5 min at room temperature. Then, the temperature of the samples was increased up to 37°C over 1 min, and the reactions were triggered by adding arachidonic acid to a final concentration of 5 μM for COX-1 and 2 μM for COX-2. After 5 min, 1 mL of ice-cold methanol was added and the reactions were blocked on ice. Internal PGB_1_ standard (183 ng) and 530 μL of acidified phosphate-buffered saline (PBS) were added for the purification step, and solid-phase extraction was performed using C18 RP-columns (100 mg, UCT, Bristol, PA, United States) as reported previously (Albert et al., 2002). COX product formation was calculated by analyzing 12- hydroxyheptadecatrienoic acid (12-HHT) formation by means of RP-HPLC (Albert et al., 2002).

### Cell Viability Assay

Cell viability was determined by MTT conversion assay. The cells were seeded in triplicate in 96 well-plates and incubated with increasing concentrations (between 5 and 100 μM) of compounds **1c**, **2c** and CAY10526 (Cayman Chemical Company), or DMSO 0.1% (v/v) for the 24, 48, or 72 h in DMEM. Following the treatment, 20 μL of MTT solution (5 mg/mL in PBS) [3-4,5-dimethyldiazol-2-yl]-2,5-diphenyl tetrazolium bromide reagent (Sigma-Aldrich), was added, and the cells were incubated for an additional 3 h at 37°C. The formazan crystals thus formed were dissolved in 100 μL of buffer containing 50% (v/v) N,N-dimethylformamide, 20% SDS (sodium dodecyl sulfate) (pH 4.5). The IC_50_ values were defined as the compound concentration resulting in 50% inhibition of cell survival compared to control cells treated with DMSO. The absorbance was measured at 570 nm with a Multiskan™ GO Microplate Spectrophotometer (Thermo Fisher Scientific, Waltham, MA, United States).

### Cell-Cycle Progression Analysis

The A549 cells were treated with compound **2c** (5 or 10 μM) or DMSO for 24, 48, or 72 h in DMEM with 10% FBS. After each treatment, the cells were harvested and incubated with propidium iodide (PI) solution (0.1% sodium citrate, 0.1% Triton X-100, and 50 mg/mL of PI) for 30 min at 4°C. For each sample, 10,000 events were recorded using FACScalibur flow cytometry (Becton Dickinson, San Jose, CA, United States), and the proportion of the cells in each cell cycle phase was calculated using the ModFit LT software (BD). Necrosis/apoptosis cell fraction was quantified as the proportion of cells with hypodiploid DNA (subG_0_/G_1_ peak) using the CellQuest software (Becton Dickinson). The data reported were obtained by two different experiments with similar results, performed in duplicate, and the statistical significance of results was examined using two-way ANOVA with Bonferroni post-test analysis.

### Statistical Analysis

The obtained data are expressed as mean ± SEM of single determinations performed in independent experiments on different days. The IC_50_ values were graphically calculated from averaged measurements at five different concentrations of the compounds by means of the GraphPad Prism 4.0 software (San Diego, CA, United States). One-way ANOVA followed by Tukey-Kramer *post-hoc* test for multiple comparisons was applied for statistical evaluation of the data. A *p* value of <0.05 (^*^) was considered significant.

## Results

### Molecular Modeling

**Figure 2:**
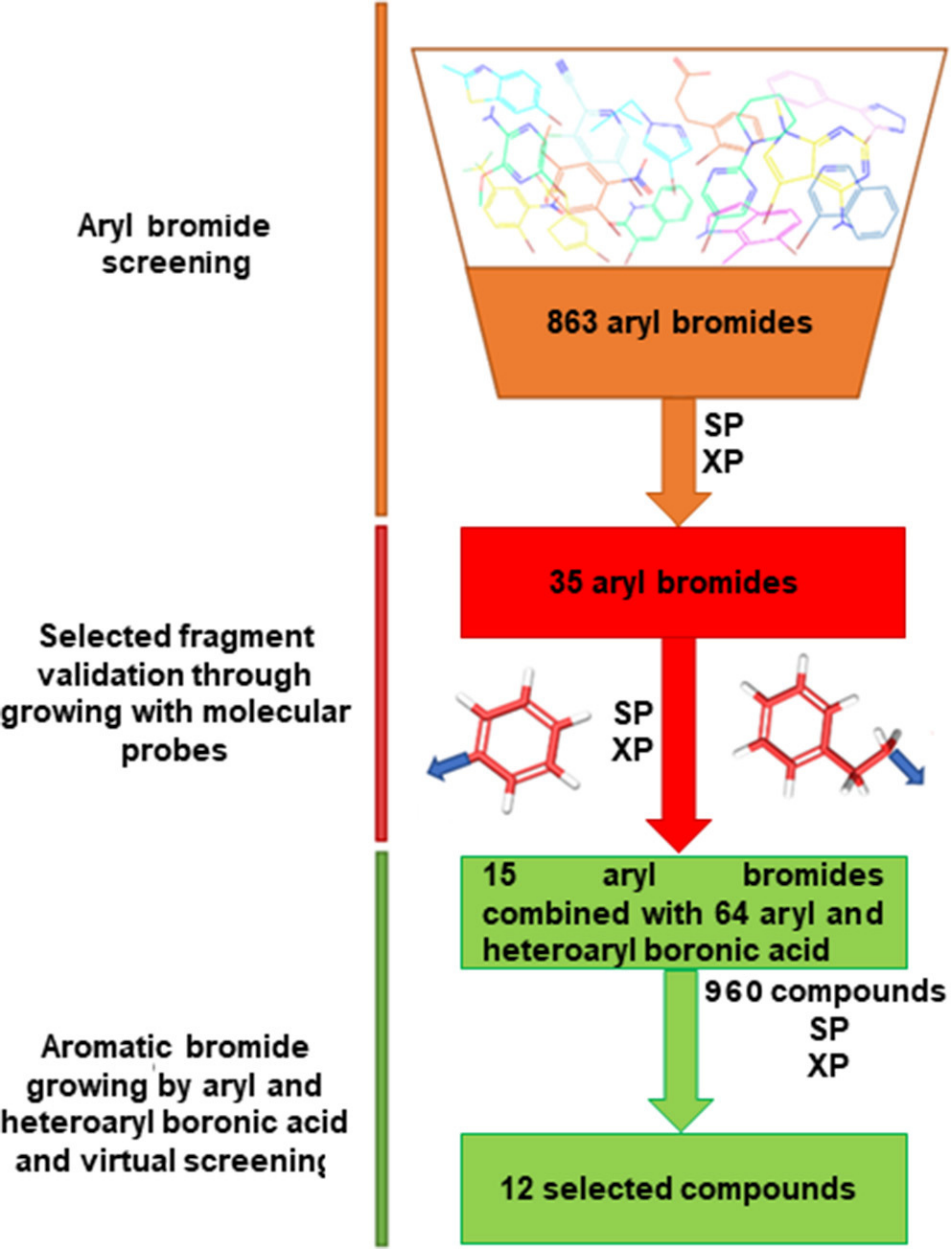
Fragment-based approach workflow.

We adopted a virtual fragment screening approach (Botta et al., 2015; Giordano et al., 2018a, 2019) starting from aromatic bromides, straightforwardly modifiable by the Suzuki-Miyaura reaction, as successfully reported in a recent contribution of the authors (Di Micco et al., 2018). We focused on aromatic fragments as starting molecular scaffold, based on our previous observation that in the 3D protein structure, different aromatic residues, such as Phe44, His53, and Tyr130, flank the mPGES-1 binding site (Lauro et al., 2016). In the present work, we updated and enlarged the collection of commercially available (Sigma Aldrich + Otava Chemicals) aromatic bromide fragments, following an implemented *in silico* workflow, which is sketched in Figure 2.

The built library of 863 fragments was virtually screened into the mPGES-1 catalytic site by means of the Glide software (Friesner et al., 2004, 2006; Halgren et al., 2004; Glide, 2017), which is a very useful tool in fragment-based drug design (Loving et al., 2009; Sándor et al., 2010; Good et al., 2012; Vass and Keseru, 2013; Lauro et al., 2016; Di Micco et al., 2018). As for the protein model, we employed the high-resolution crystal structure (1.16 Å) of the enzyme linked to GSH (PDB ID: 4AL0) (Sjçgren et al., 2013), as no significant structural changes upon inhibitor binding were observed by comparing the different reported experimental structures (Li et al., 2014; Luz et al., 2015; Kuklish et al., 2016; Schiffler et al., 2016; Partridge et al., 2017) of mPGES-1. In our analysis of docked fragments into the catalytic cavity, we particularly paid attention to the bromine atom orientation, representing the functionalization point according to the synthetic strategy used. The docking outcomes suggested 35 potential molecular seeds that can be employed for chemical modifications, all showing to make interactions with residues Phe44, Asp49, Arg52, His53, Arg126, and Ser127. These fragment candidates were expanded by substituting the bromine with both an aryl and a phenylethyl moiety as molecular probes mimicking the boronic acid partners into Suzuki reactions, to evaluate their influence on the binding mode of the parent molecular seeds (Figure 2). The so-built 70 small molecules were subjected to virtual screening, selecting 15 potential aromatic bromides as electrophile partners in the Suzuki-Miyaura reaction. The docked poses of the selected fragments showed that the bromine atom points toward a 9 Å cavity delimited by the transmembrane helixes (Sjçgren et al., 2013; Di Micco et al., 2016, 2018), constituted by the residues A:Tyr28, A:Ala31, A:Ile32, A:Gln36, C:Ser127, C:Tyr130, C:Thr131, C:Gln134, C:Leu135, and C:Cys137. Hence, we combined the selected fragments with 64 boronic acids endowed with two aromatic rings to cover the unoccupied 9-Å pocket and to establish π-π interactions, van der Waals contacts, and H-bonds with the macromolecular counterparts. These structural considerations are fully supported by x-ray crystallography (Sjçgren et al., 2013; Li et al., 2014; Luz et al., 2015; Kuklish et al., 2016; Schiffler et al., 2016; Partridge et al., 2017), showing that the inhibitors bound to mPGES-1 give π-π interactions with Tyr28 and Tyr130 and van der Waals contacts with the other residues outlining the transmembrane cavity. The 960 generated compounds were screened by molecular docking and the obtained outcomes narrowed down the library to, at least, two small collections of compounds **1a**-**1f** and **2a**-**2f** (Figure 2), which are helpful for grasping a basic structure–activity profile.

**Figure 3:**
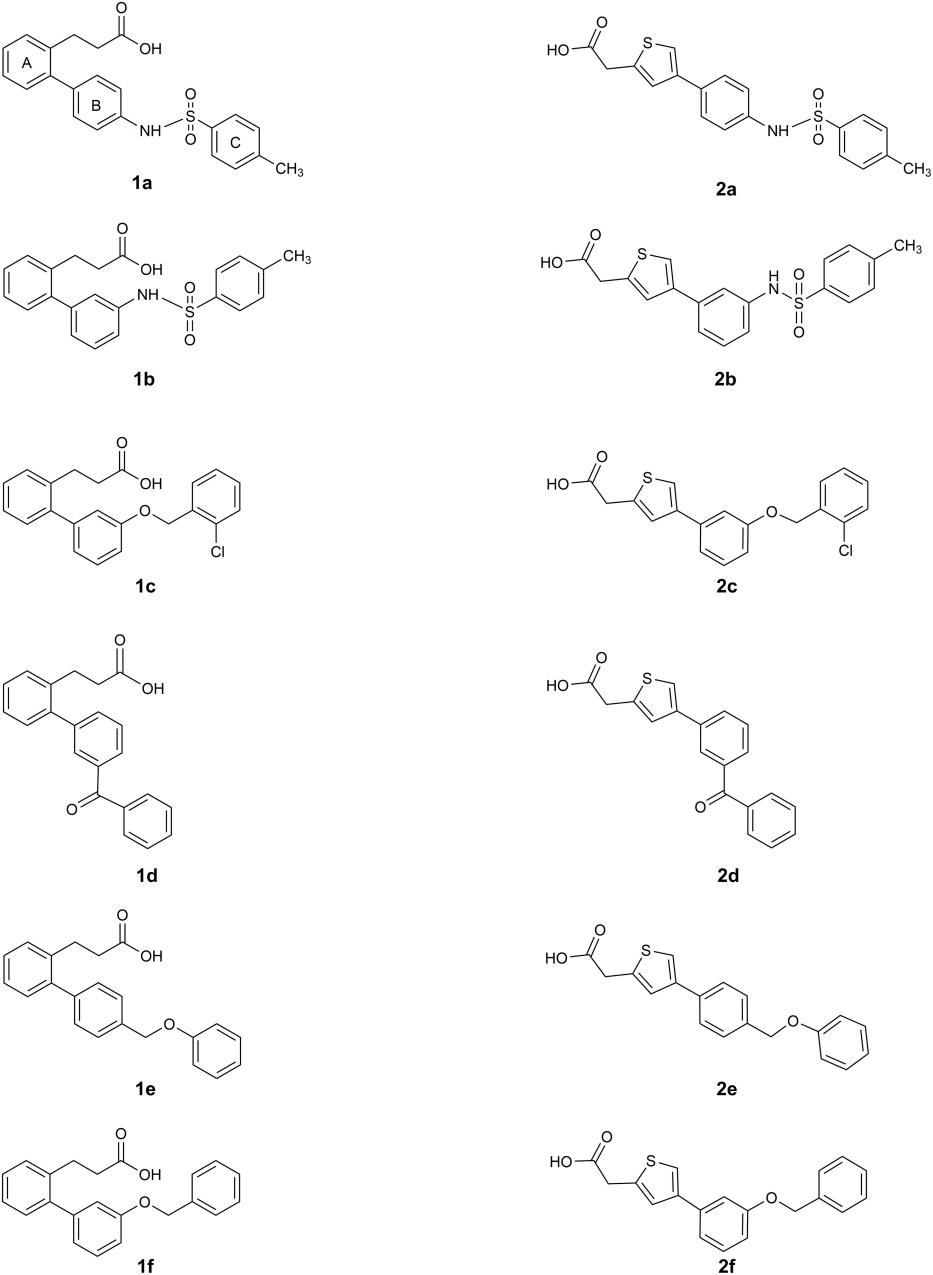
Molecular structure of compounds **1a-f** and **2a-f**.

**Figure 4:**
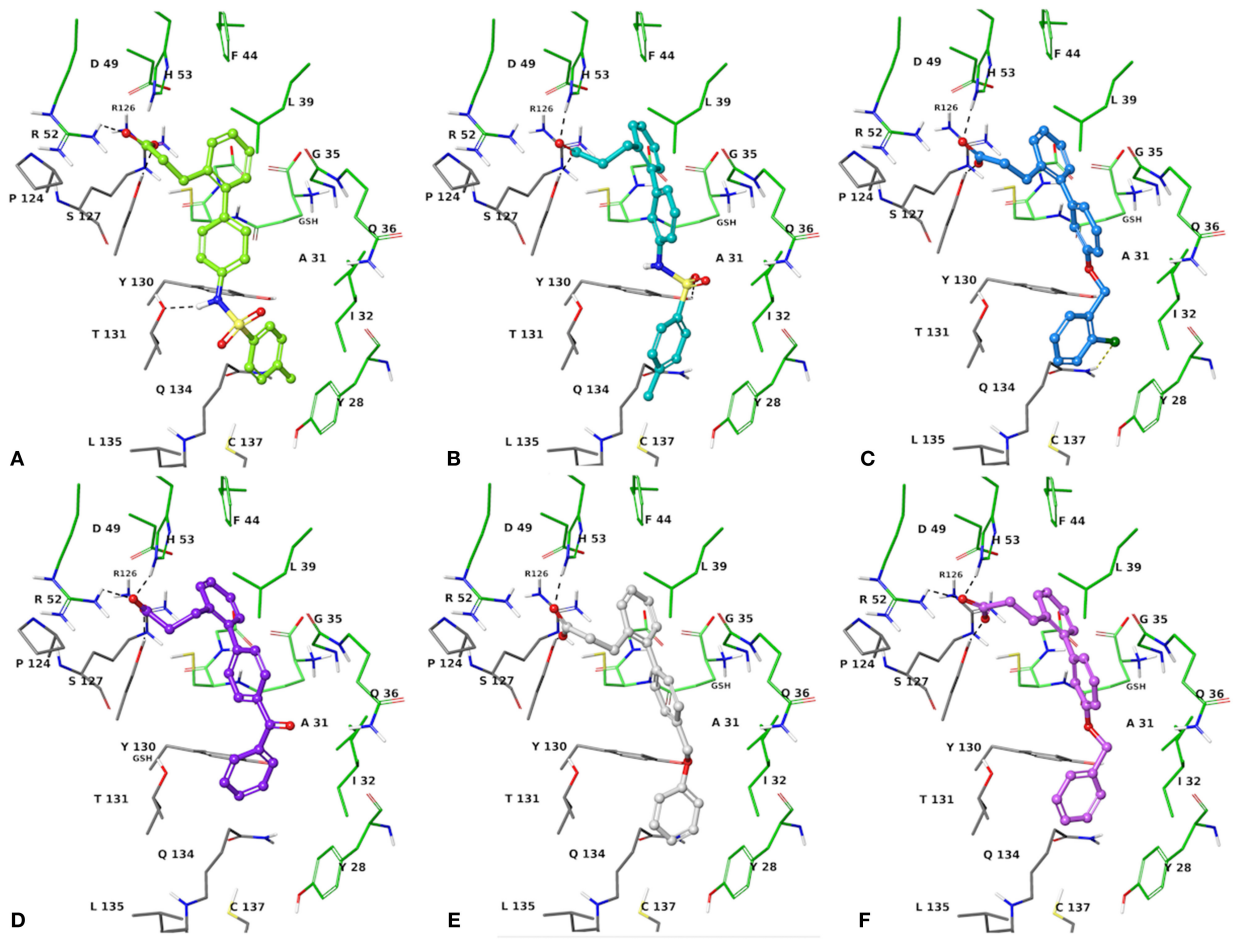
Three-dimensional model of the interactions given by **1a (A)**, **1b (B)**, **1c (C)**, **1d (D)**, **1e (E)**, and **1f (F)** with mPGES-1. The protein is depicted by tube [colored: C, gray (chain A) and green (chain B); polar H, white; N, dark-blue; O, red; S, yellow]. The GSH is sketched in the faded green tube. The small molecules are represented by sticks (yellow-green for **1a**, teal for **1b**, azure for **1c**, violet for **1d**, platinum for **1e**, faded plum for **1f**) and balls (colored: C, as for the sticks; polar H, white; N, dark-blue; O, red). The dashed black and yellow lines indicate the hydrogen and halogen bonds, respectively, between ligand and protein.

**Figure 5:**
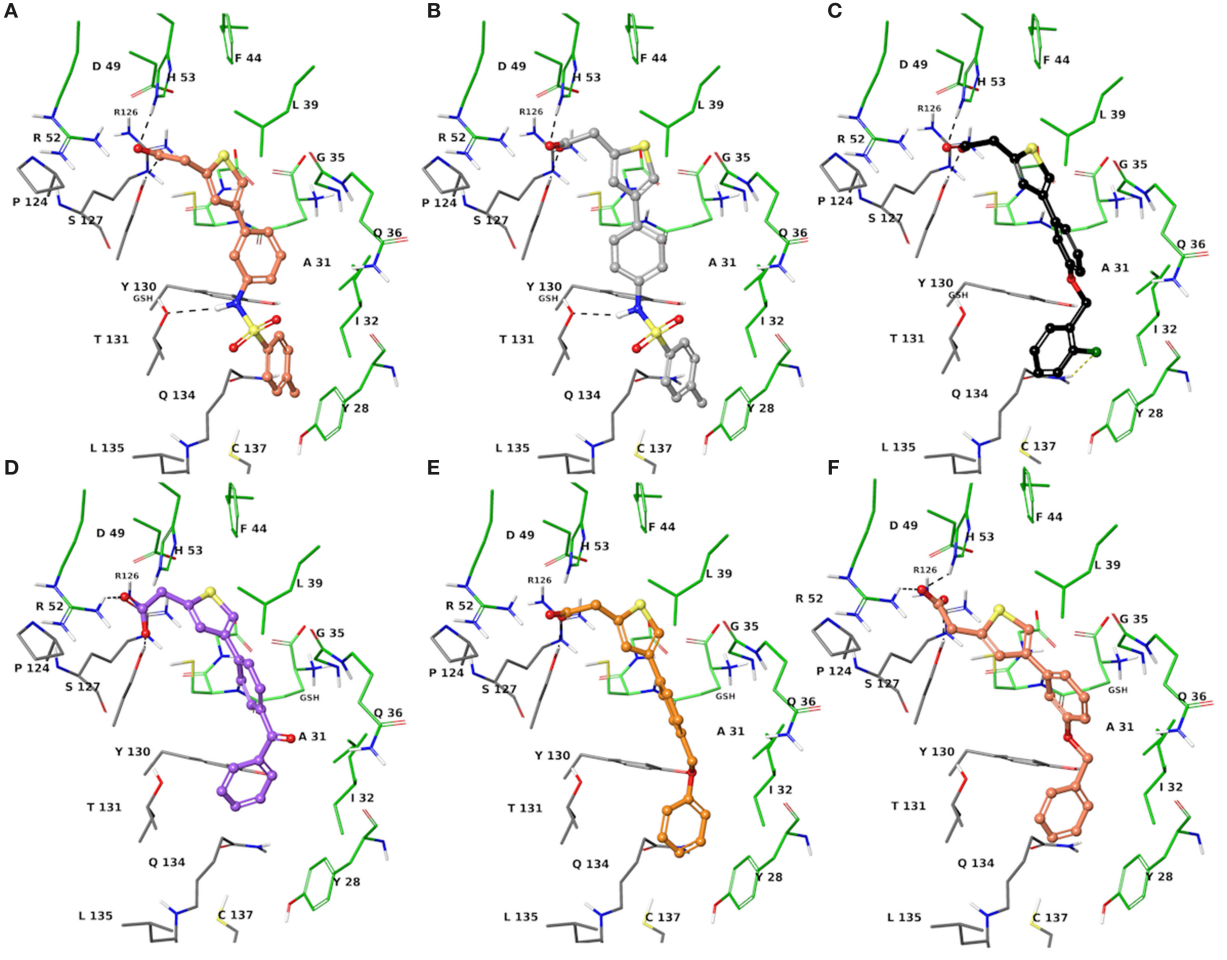
Three-dimensional model of the interactions given by **2a (A)**, **2b (B)**, **2c (C)**, **2d (D)**, **2e (E)**, and **2f (F)** with mPGES-1. The protein is depicted by the tube [colored: C, gray (chain A) and green (chain B); polar H, white; N, dark-blue; O, red; S, yellow]. The GSH is sketched in the faded green tube. The small molecules are represented by sticks (faded salmon for **2a**, light gray for **2b**, black for **2c**, faded violet for **2d**, orange for **2e**, faded red-orange for **2f**) and balls (colored: C, as for the sticks; polar H, white; N, dark-blue; O, red). The dashed black and yellow lines indicate the hydrogen and halogen bonds, respectively, between ligand and protein.

The two sets of compounds **1a**-**f** and **2a-f** contain as basic units, respectively, a 3-biphenyl-2-yl propionic acid and a 4-phenyl-(thiophen-2-yl)acetic acid, derived from the starting aromatic bromide fragments coupled with six different boronic acids (Figure 3). The analysis of the docked poses of **1a**-**f** and **2a**-**f** (Figures 4, 5) showed that all the compounds fill equivalent spaces of the catalytic pocket, making a similar pattern of interactions with the protein counterparts (Table 1). In detail, they establish H-bonds with Arg52, His53, and Ser127, whereas ring A gives a π-cation with Arg126 and an aromatic H-bond with Asp49 (Figures 4, 5). It is worth noting that the contacts with residues Arg52, His53, and Ser127 have been already shown by co-crystallized binders of mPGES-1 (Li et al., 2014; Luz et al., 2015; Kuklish et al., 2016; Schiffler et al., 2016; Partridge et al., 2017). The 3-phenylpropanoic acid (**1a**-**f**) and the 2-(thiophen-2-yl)acetic acid (**2a**-**f**) portions also establish van der Waals contacts with Gly35, Leu39, Phe44, Arg52, His53, Ala123, and Pro124 (Figures 4, 5). The central aromatic ring gives π-π interactions with Tyr130 and contacts with Gly35, Ser127, Tyr130, and Thr131 (Figures 4, 5). The ring C is engaged in π-π interactions with Tyr130 and gives van der Waals bonds with Tyr28, Ile32, Thr131, and Gln134 (Figures 4, 5). Compounds **1d** and **2d**, compared to their congeners, do not give contacts with Tyr28 and Gln134 (Figures 4, 5). The sulphonamide groups in **1a**, **2a**, and **2b** donate an H-bond to Thr131 (Figures 4A, 5A,B), whereas **1b** accepts an H-bond from Tyr130 (Figure 4B). The oxymethylene group of **1c**, **1e**, **1f**, **2c**, **2e**, and **2f** is hydrogen-bonded to the side chain of Tyr130 (Figures 4C,E,F, 5C,E,F). In this extensive network of contacts, the halogen bond, established by the chlorine atom present on ring C of **1c** and **2c**, could be responsible for the higher affinity with the protein model and accordingly for the higher biological activity manifested.

**Table 1:** Docking scores for **1a**-**f** and **2a**-**f**.

**Compound**	**Docking score (kcal/mol)**
**1a**	−8.144
**1b**	−7.694
**1c**	−8.307
**1d**	−7.768
**1e**	−8.236
**1f**	−8.314
**2a**	−7.929
**2b**	−7.185
**2c**	−8.736
**2d**	−7.300
**2e**	−8.627
**2f**	−6.415

### Chemical Synthesis

**Scheme 1:**
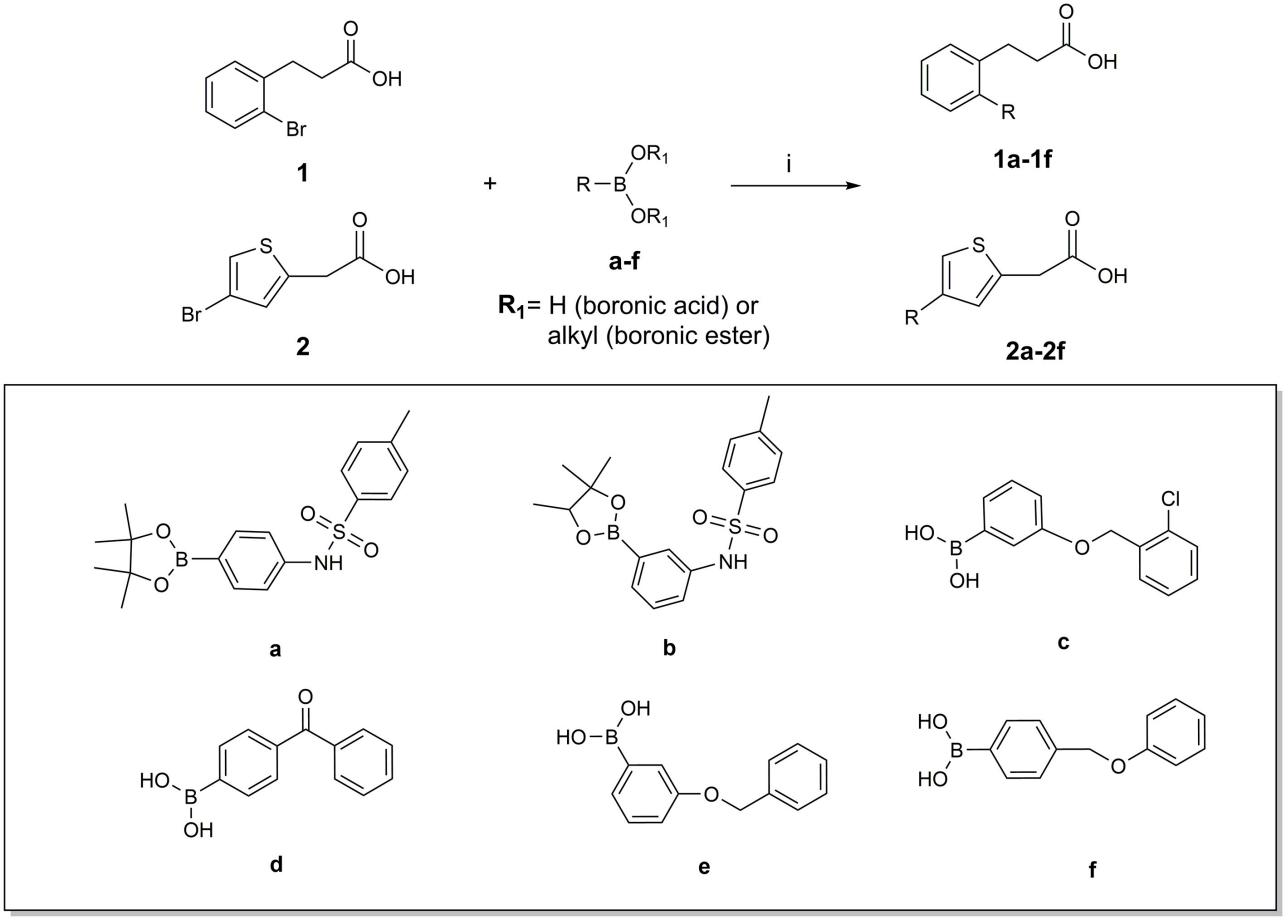
Synthetic procedure: (i) Pd(PPh_3_)_4_, K2CO3, dioxane/H_2_O (2:1), reflux, overnight.

The synthesis of compounds **1a-f** and **2a-f** was accomplished by the Suzuki-Miyaura cross-coupling reaction of 3-(2-bromo-phenyl)-propionic acid **1** or (4-bromo-thiophen-2-yl)-acetic acid **2** with selected arylboronic acids/esters (**a**-**f**), bearing various substitution patterns, as reported in Scheme 1. By means of the conventional Suzuki reaction conditions, the designed compounds **1a-f** and **2a-f** were easily prepared with good and moderate yields, respectively, and under mild conditions (see Experimental section) (Miyaura and Suzuki, 1995; Di Micco et al., 2018; Campeau and Hazari, 2019).

### Biological Investigation

**Figure 6:**
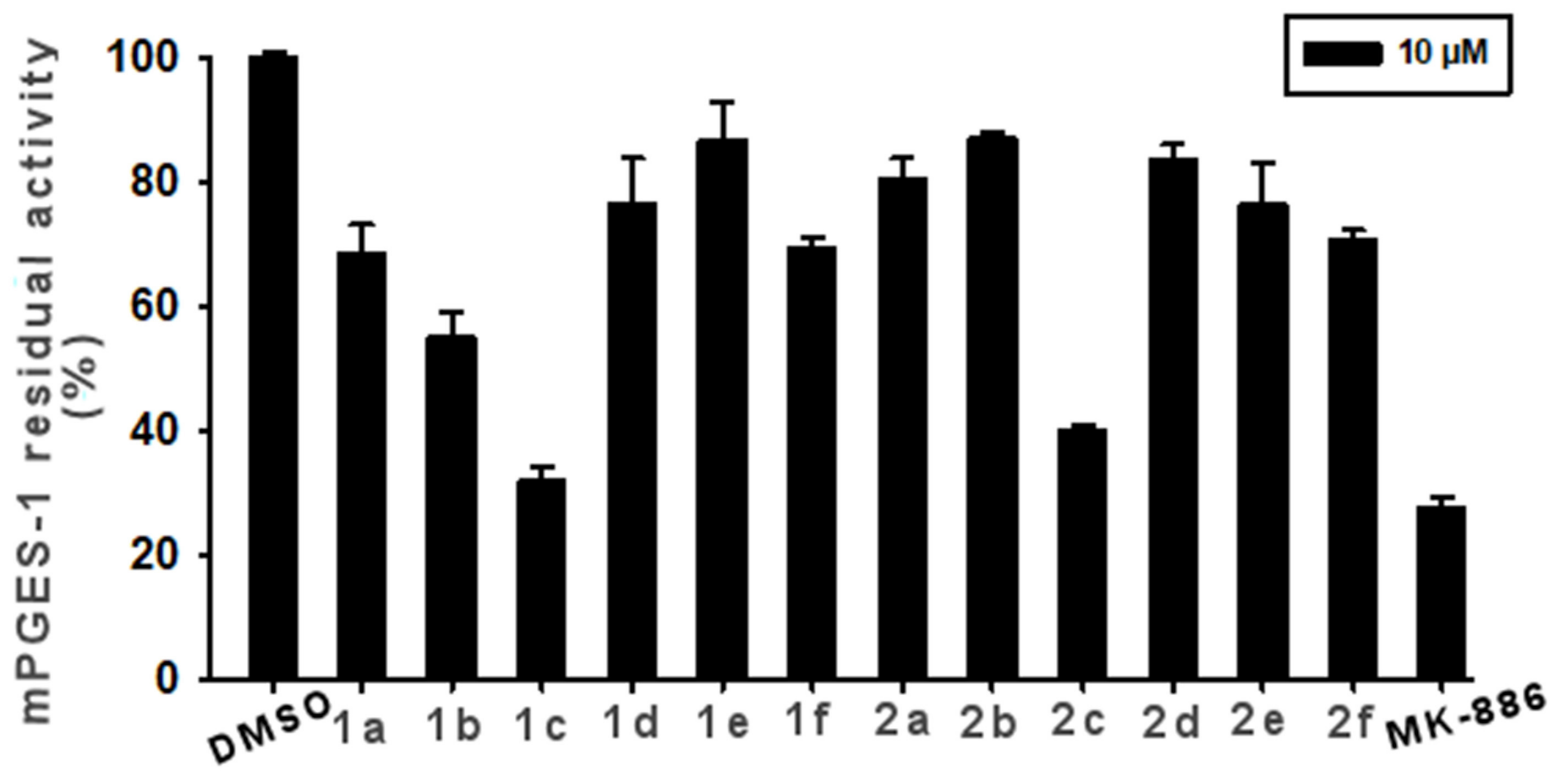
Inhibition of mPGES-1 by compounds **1a**-**f**, **2a**-**f**, and MK-886 (10 μM) in a cell-free enzyme activity assay. The percentage of residual mPGES-1 activity is calculated with respect to the control. Data are given as mean ± SEM, *n* = 3.

The synthesized compounds (**1a-f** and **2a-f**) were investigated for inhibition of human mPGES-1, derived from microsomes of the IL-1β-stimulated A549 cell line by means of cell-free assay. All compounds were tested at 10 μM and the residual mPGES-1 activity was detected as shown in Figure 6.

The experiments showed that **1c** and **2c** presented the highest inhibitory activity (>60%, Figure 6). Compound **1b** also appreciably decreased enzyme activity by 42%. Lower enzyme modulation (20–25%) was observed for **1a**, **1f**, **2e**, and **2f**, whereas the remaining analogs (**1d**, **1e**, **2a**, **2b**, and **2d**) presented an inhibitory activity of ≤ 20%. Notably, these experimental data are in good qualitative agreement with the theoretical outcomes. Indeed, the chlorine, atom present on ring C and its specific spatial position on ring B, contributes to the increase in binding affinity for the target enzyme.

We further proceeded to investigate the inhibitory activity of **1c** and **2c** by evaluating their IC_50_ values *vs*. mPGES-1 through the same assay. Comparable IC_50_ values in the low micromolar range were observed for compounds **1c** (IC_50_ = 3.4 ± 0.5 μM) and **2c** (IC_50_ = 5.9 ± 1.0 μM).

**Figure 7:**
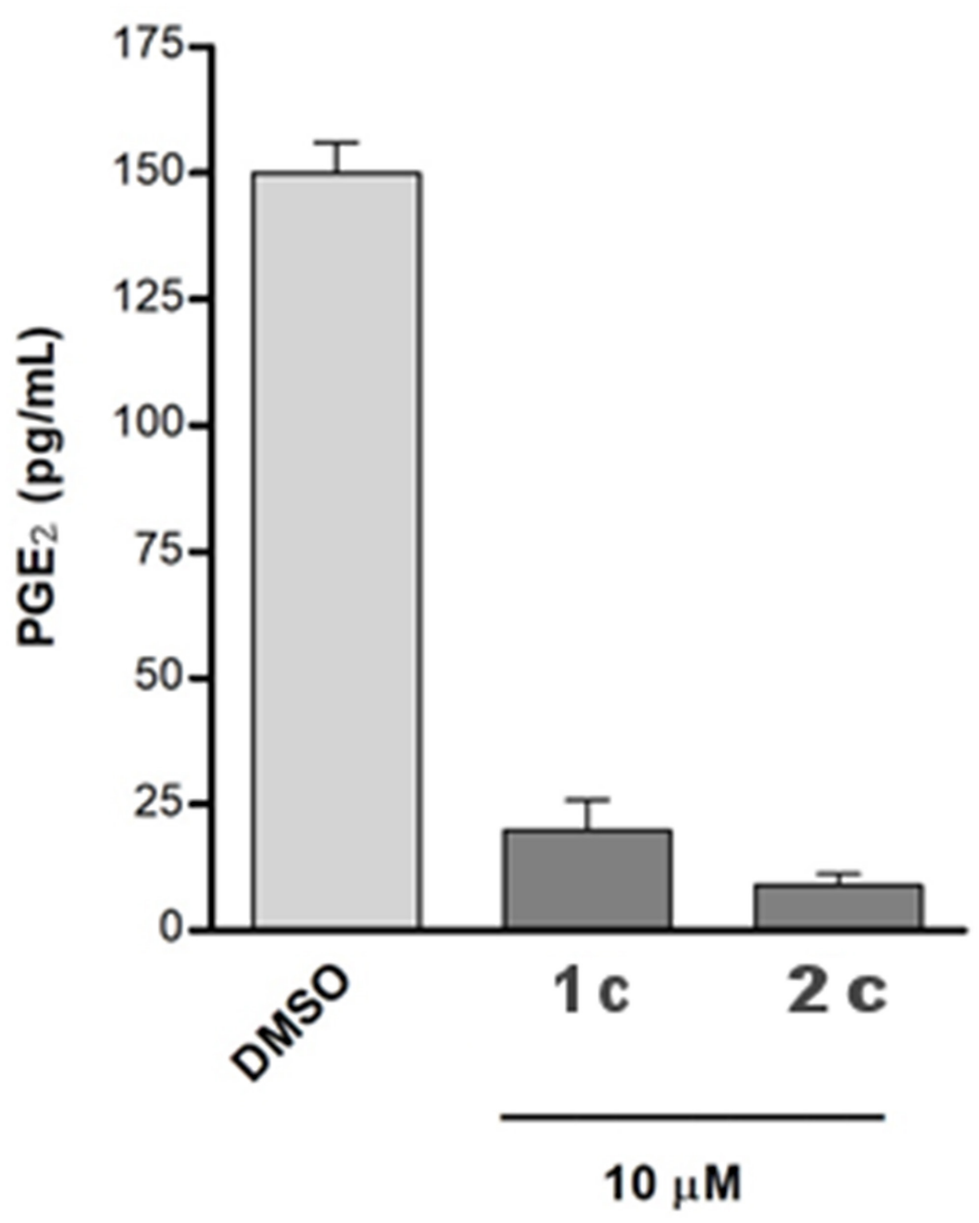
The A549 cells were incubated for 24 h with 10 μM of compounds in conditioned medium (1% FBS and 10 ng/mL IL-1β). The PGE_2_ released into medium was quantified using a specific ELISA kit assay. The results were compared with that of the control cells IL-1β stimulated and treated with chemical vehicle (DMSO) and are expressed as mean ± SEM (pg/mL) of two different experiments.

Moreover, we investigated the effects of compounds **1c** and **2c** on PGE_2_ production in A549 cells, which were incubated with IL-1β and the two selected compounds (10 μM) for 24 h. Both the tested molecules considerably reduced cytokine-induced PGE_2_ production (Figure 7).

**Figure 8:**
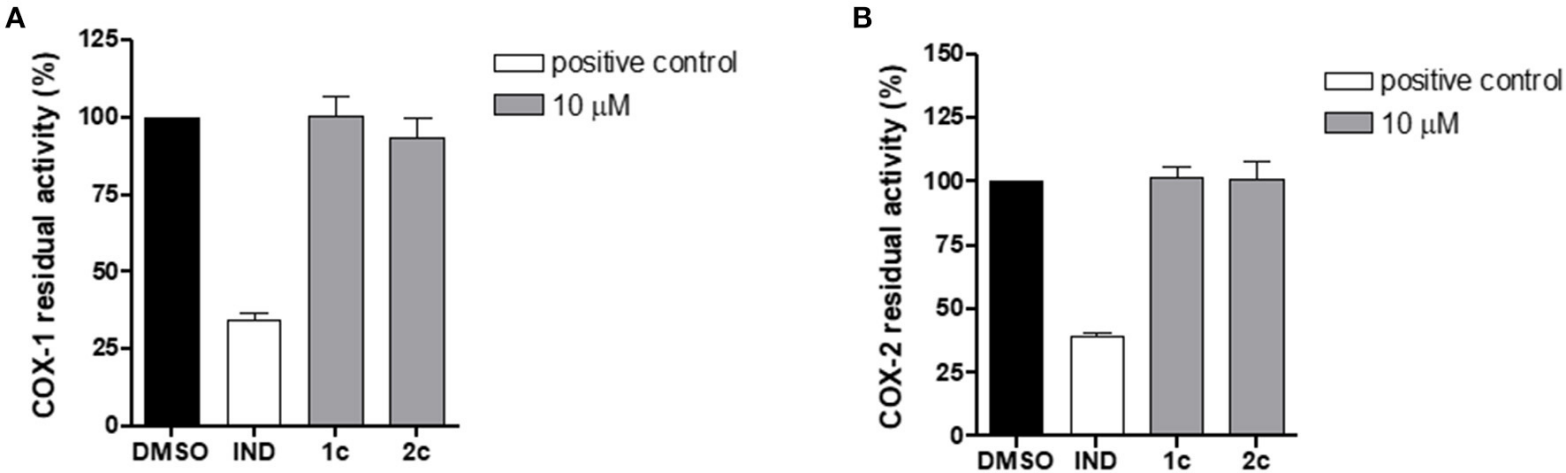
Residual activity of COX-1 **(A)** and COX-2 **(B)** after incubation with compounds **1c**, **2c**, and indometacin (10 μM) or vehicle (0.1% DMSO). Data are expressed as a percentage of control (100%), *n* = 3.

To assess the selectivity of the compounds, we investigated the impact of **1c** and **2c** on both COX isoforms by means of cell-free assays to exclude possible effects on other prostanoid formations. Specifically, the enzymes were incubated with **1c** and **2c** at 10 μM or vehicle (0.1% DMSO) and the 12-HHT formation was quantified. Interestingly, the compounds did not compromise the activity of COXs (Figure 8), thus confirming their selective inhibition against mPGES-1, which is known to be functionally linked to the inducible isoform COX-2 on the arachidonic acid cascade.

### Cytotoxic Activity and Cell Cycle

Microsomal prostaglandin E synthase-1 is highly expressed and strongly associated with signaling pathways in different types of malignant cells. As suggested in previous studies, the decreasing of mPGES-1 activity may inhibit cell proliferation, induce apoptosis, and cell cycle arrest.

In order to evaluate the potential anticancer properties of the mPGES-1 inhibitors, we tested the antiproliferative or cytotoxic effect of compounds **1c** and **2c** (5–100 μM) in human A549 cells (Hanaka et al., 2009; Maeng et al., 2014) after exposure for 24, 48, and 72 h, determining cell viability by MTT proliferation assay. The compound CAY10526, a selective modulator of mPGES-1 expression (Guerrero et al., 2007), was used as control in our experiments and reproduced the previously reported IC_50_ values well (Chini et al., 2020). Moreover, the IC_50_ values at 48 and 72 h, obtained for CAY10526, were in agreement with the concentrations used to evaluate the interference of the PGE_2_ production levels in A549 cells (Maeng et al., 2014). As shown in Table 2, **1c** and **2c** inhibited the cancer cell vitality in the A549 cell line, exhibiting good cytotoxic activity compared to the known inhibitor (CAY10526). Since compound **2c** showed the best IC_50_ values after 48 and 72 h of exposure, it was selected for further analysis.

**Table 2:** Cell viability assay of mPGES-1 inhibitors in the A549 cancer cell line.

**Compound**		**IC_**50**_ (μM)**	
	**24 h**	**48 h**	**72 h**
**1c**	90.3 ± 1.5	48.2 ± 1.1	36.4 ± 1.5
**2c**	65.5 ± 1.2	18.1 ± 0.9	10.1 ± 1.2
CAY10526	62.5 ± 0.5	12.5 ± 1.1	9.1 ± 1.3

*IC_50_ (μM) values are given as mean ± SEM of single determinations obtained from three independent experiments at different exposures (24, 48, 72 h)*.

**Figure 9:**
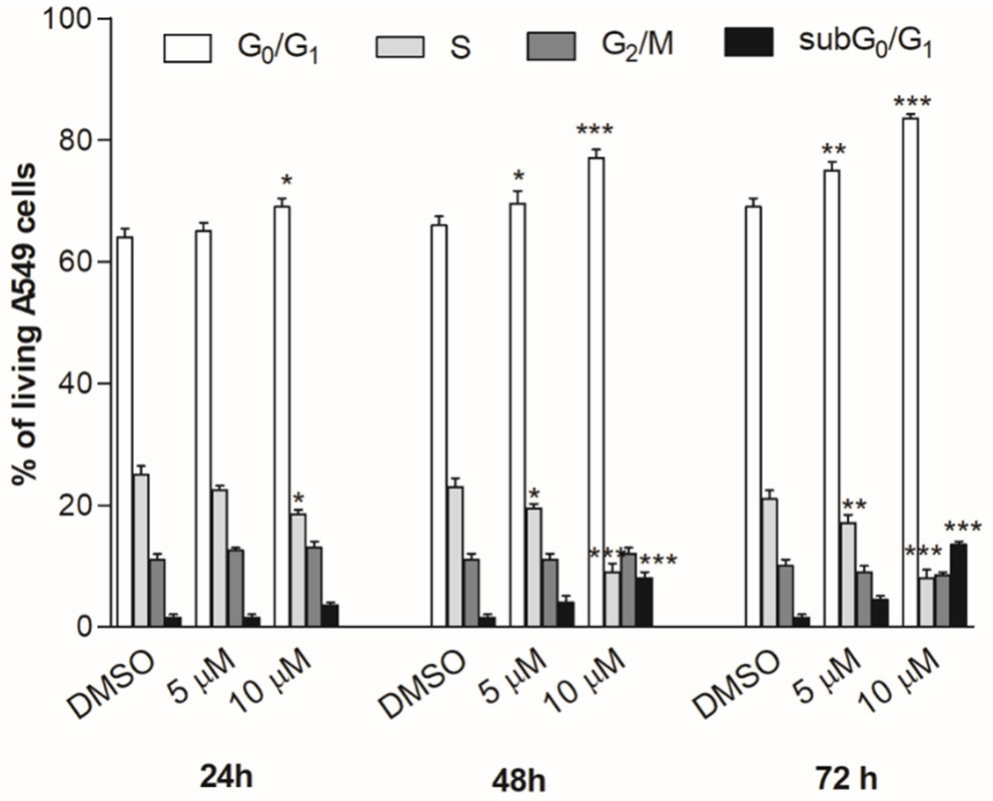
Cell cycle progression. A549 cancer cells were treated for 24, 48, or 72 h with compound **2c** used in different concentrations. The percentage of cell cycle stages was analyzed by flow cytometry with DNA propidium iodide staining. Results are expressed as mean ± SEM of two independent experiments performed in triplicate (*P < 0.05, **P < 0.005, ***P < 0.001).

The cell cycle progression analysis of A549 cancer cells by flow cytometry was performed to explore the effect of compound **2c** on the inhibition of cancer cell viability, using concentrations close to the best IC_50_ values (Table 2). The treatment with **2c** displayed a modest cytostatic activity (Figure 9) after 24 h, and this effect was enhanced after 48 and 72 h with a considerable arrest in the G_0_/G_1_ phase. Moreover, the cell exposure with 10 μM of **2c** for 48 and 72 h caused an increase in subG_0_/G_1_ fraction (about 8.0 ± 0.9% and 13.5 ± 0.5%, respectively), suggesting the occurrence of an apoptosis/necrosis event.

## Discussion

By combining a virtual fragment-based approach and biological investigations in cell-free and cell-based assays, we succeeded in identifying the 4-phenyl-(thiophen-2-yl)acetic acid-based compound **2c** as a novel lead compound worthy to progressively develop new-generation m-PGES-1 inhibitors with anti-tumoral and/or anti-inflammatory properties. Compound **2c** showed low micromolar activity against mPGES-1 and did not affect COXs in cell-free assays. Considering the well-known side effects of commercially available COX inhibitors, the selectivity for mPGES-1 of compound **2c** is a promising outcome that paves the way to further investigation. Interestingly, **2c** demonstrated considerable inhibition of PGE_2_ production in A549 cells. On the same cell line, **2c** showed good cytotoxic activity.

In particular, we observed that the biological activity profile of **2c** at 24, 48, and 72 h is comparable to the reference compound, CAY10526. Interesting results were also obtained for **1c**, which presents a selective inhibition activity of mPGES-1 in the low micromolar range. Moreover, **1c** considerably inhibits the PGE_2_ biosynthesis in A549 cells similarly to **2c**, and its cytotoxicity is lower than that of **2c**. However, the 4-phenyl-(thiophen-2-yl)acetic acid-based scaffold could be used as the molecular framework for the rational design of new mPGES-1 inhibitors. The structure–activity relationships gathered in our investigations suggested that ring C and its *meta* position on ring B are responsible for the increase in the binding affinity toward mPGES-1. In particular, the presence of a chlorine atom on both the most active compounds against mPGES-1 (**1c** and **2c**) highlights the advantage of a halogen bond for strengthening the ligand–protein complex formation. Furthermore, rings B and C greatly contribute to the complex line-up through π-π interactions, van der Waals contacts, and H-bonds with transmembrane residues. The aryl bromide-derived portion also contributes to intermolecular recognition by contacts with Arg52, His53, and Ser127. The experimental outcomes validated the implementation of our *in silico* pipeline, which uses phenyl and phenylethyl groups as molecular probes to mimic the boronic acid partner of Suzuki-Miyaura cross-coupling in order to better filter the aryl-bromides. Moreover, the enrichment of the input library collection of aryl bromides with respect to our previous work has increased the chance to explore the chemical diversity of selected molecular seeds to develop potential clinical candidates. In future works, the computer-aided approach defined in this contribution could be of great help to design tighter binders of mPGES-1 with anti-inflammatory and anti-cancer properties, and it could be adapted to different biological targets under investigation. We envisage an application toward other members of the membrane-associated proteins in eicosanoid and glutathione (MAPEG) metabolism superfamily, such as LTC_4_ and FLAP, aimed at a multitargeted approach against the prostaglandin pathway for safer therapeutical treatment. These outcomes could provide with further chances of disclosing an interesting hit to be directed toward further investigations and for adding another piece to tackle the hard challenge to develop clinical candidates. Thus, the identified lead compounds shall be tested against mouse cell lines (RAW 264.7 and NIH cells) to verify the cross-species activity, thus laying the foundation for successful *in vivo* tests.

## Dedication

This work was dedicated to the memory of our dear colleague Prof. Maurizio Botta.

## Data Availability Statement

The original contributions presented in the study are included in the article/Supplementary Materials, further inquiries can be directed to the corresponding authors.

## Author Contributions

SD and GB: conceptualization, supervision, and project administration. SD: methodology, validation, and visualization. DR and ST: synthesis. MV, MP and KF: biological assays. SD and ST: formal analysis, investigation, and data curation. GB: resources and funding acquisition. SD, ST, IB, and GB: writing—original draft preparation. SD, ST, OW, IB, and GB: writing—review and editing. All authors have read and agreed on the published version of the manuscript.

## Conflict of Interest

The authors declare that the research was conducted in the absence of any commercial or financial relationships that could be construed as a potential conflict of interest. The reviewer JL declared a past co-authorship with one of the authors OW to the handling Editor.
