# Supplementary material for: Identification of 2-(thiophen-2-yl)acetic Acid-Based Lead Compound for mPGES-1 Inhibition
Source: Front Chem. 2021 May 7;9:676631. doi: 10.3389/fchem.2021.676631 (PMC8144515; doi:10.3389/fchem.2021.676631)
Supplement: Supplementary file 1 [file Data_Sheet_1.PDF]

Supplementary Material

Identification of 2-(thiophen-2-yl) acetic acid-based lead compound for mPGES-1 Inhibition

Simone Di Micco[1], Stefania Terracciano[2], Dafne Ruggiero[2], Marianna Potenza[2], Maria C. Vaccaro[2], Katrin Fischer[3], Oliver Werz[3], Ines Bruno[2,*], Giuseppe Bifulco[2,*]

[1]European Biomedical Research Institute of Salerno (EBRIS), Via Salvatore De Renzi 50, 84125 Salerno, Italy

[2]Dipartimento di Farmacia, Università degli Studi di Salerno, Via Giovanni Paolo II 132, 84084 Fisciano (SA), Italy

[3]Institute of Pharmacy, Friedrich-Schiller-University Jena, Philosophenweg 14, D-7743 Jena, Germany

*** Correspondence:**
Giuseppe Bifulco
bifulco@unisa.it

Ines Bruno
brunoin@unisa.it

Dedicated to the memory of our dear colleague Prof. Maurizio Botta

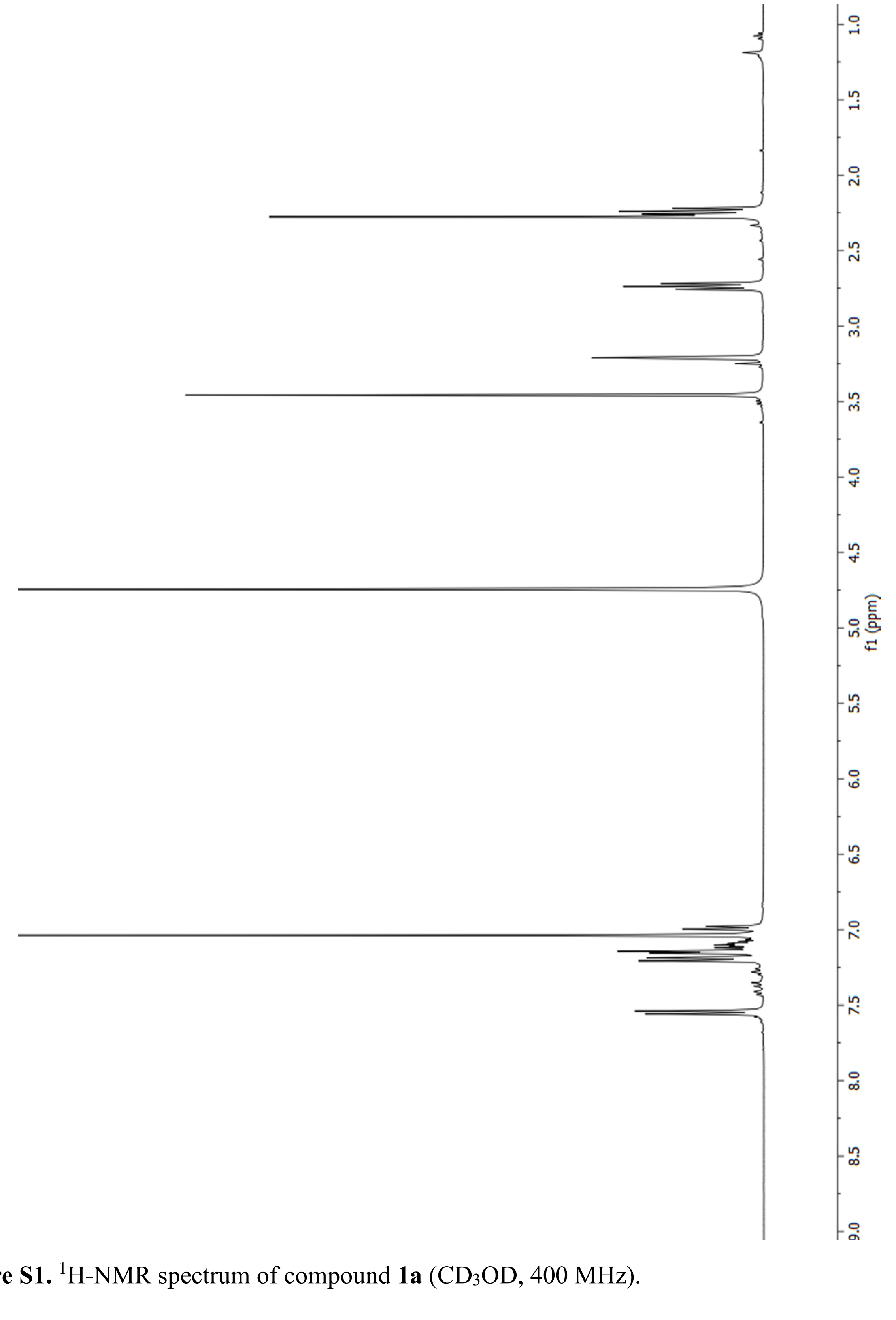

Figure S1. ^{1}H-NMR spectrum of compound **1a** (CD_3OD, 400 MHz).

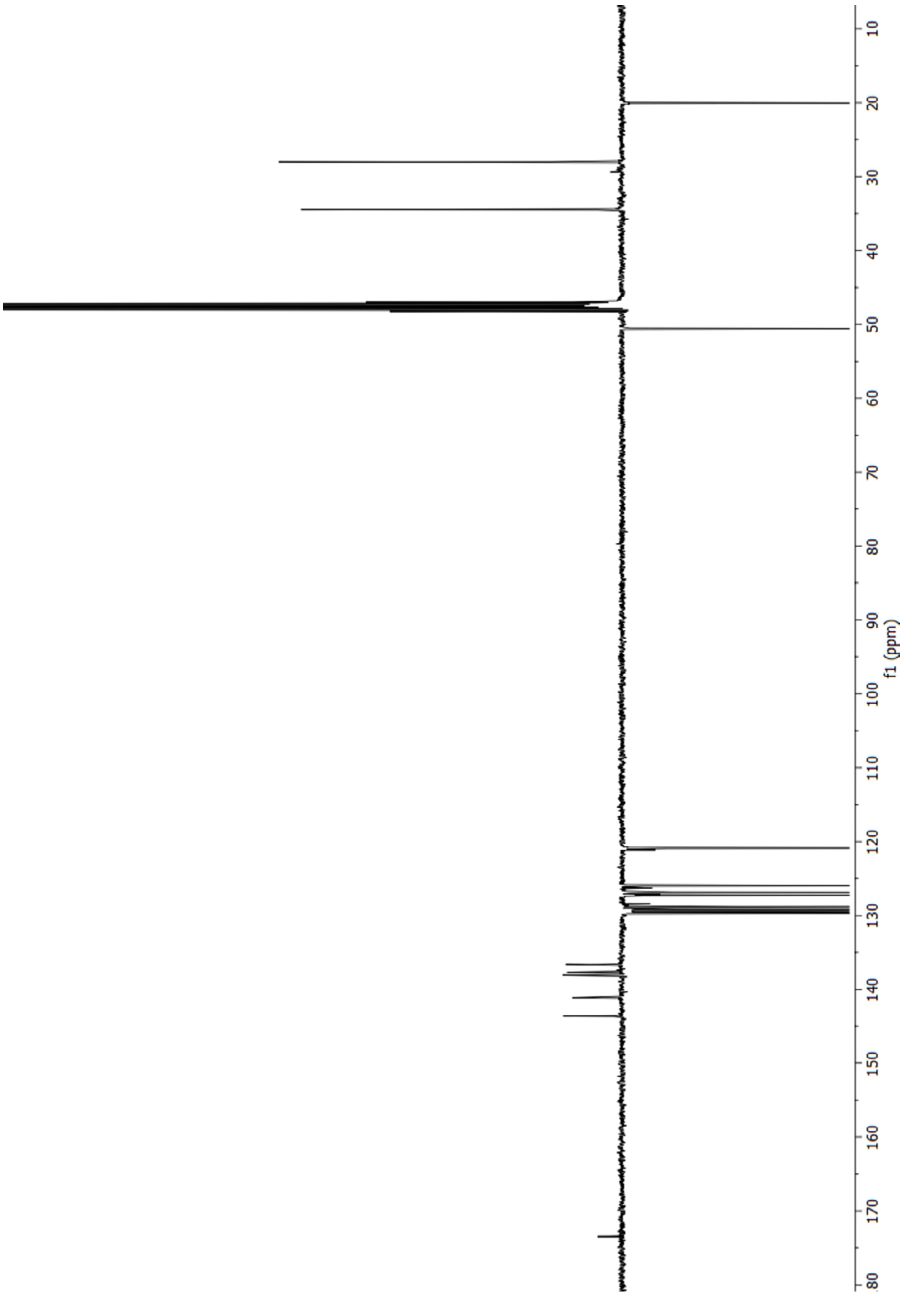

Figure S2. ^{13}C qDEPT NMR of compound **1a** (CD_3OD, 100 MHz).

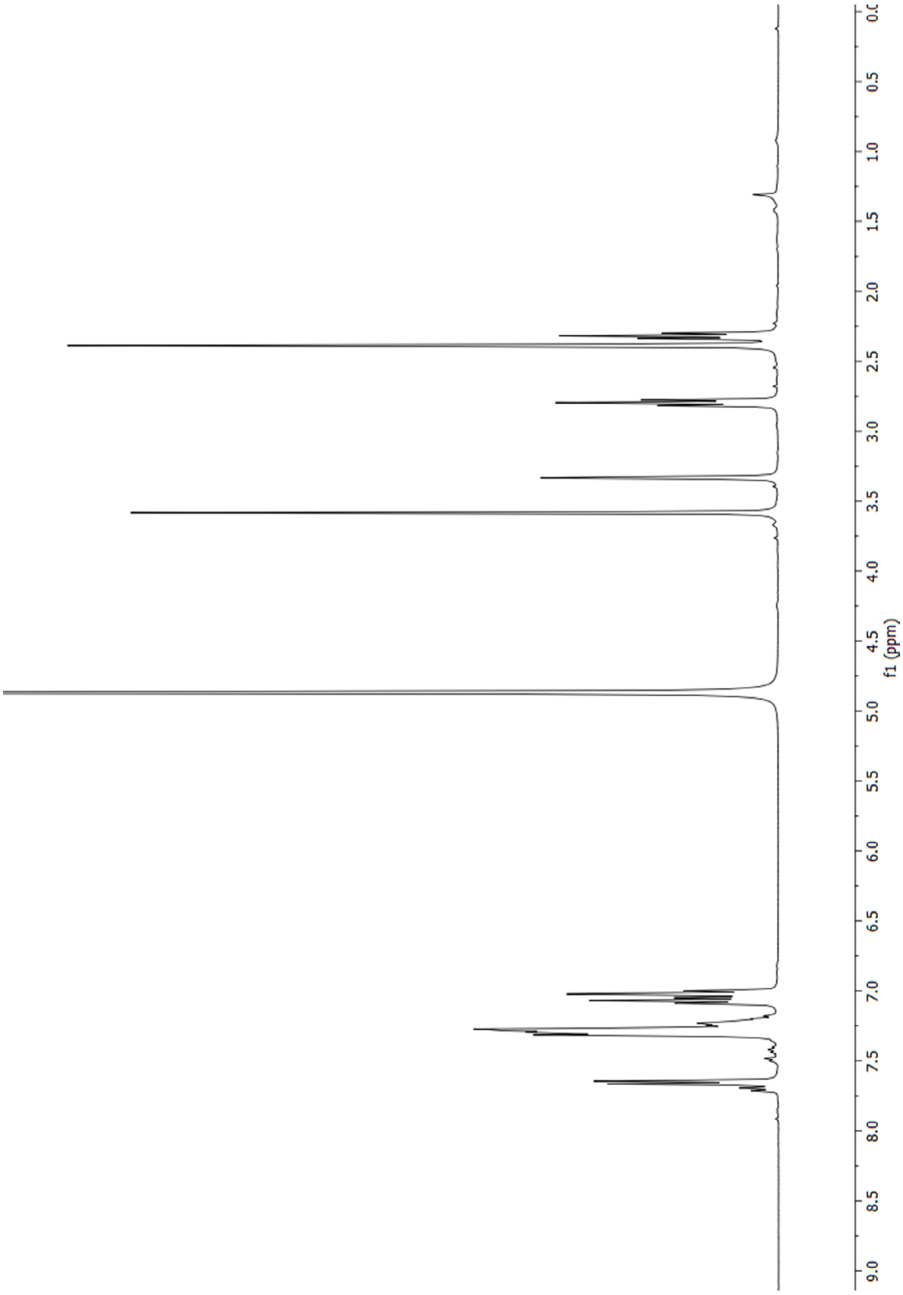

Figure S3. ^{1}H-NMR spectrum of compound **1b** (CD_3OD, 400 MHz).

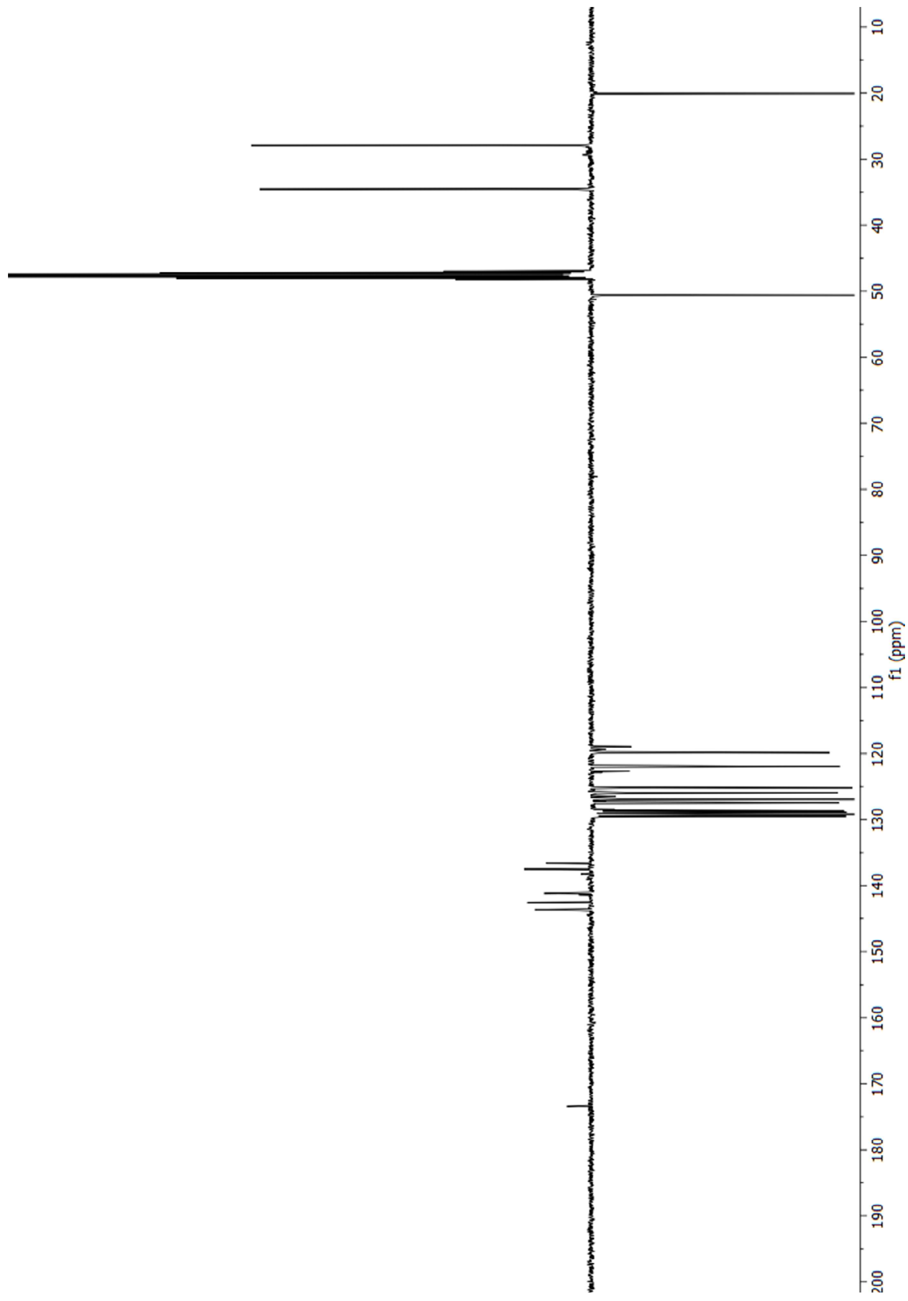

Figure S4. ^{13}C qDEPT NMR of compound **1b** (CD_3OD, 100 MHz).

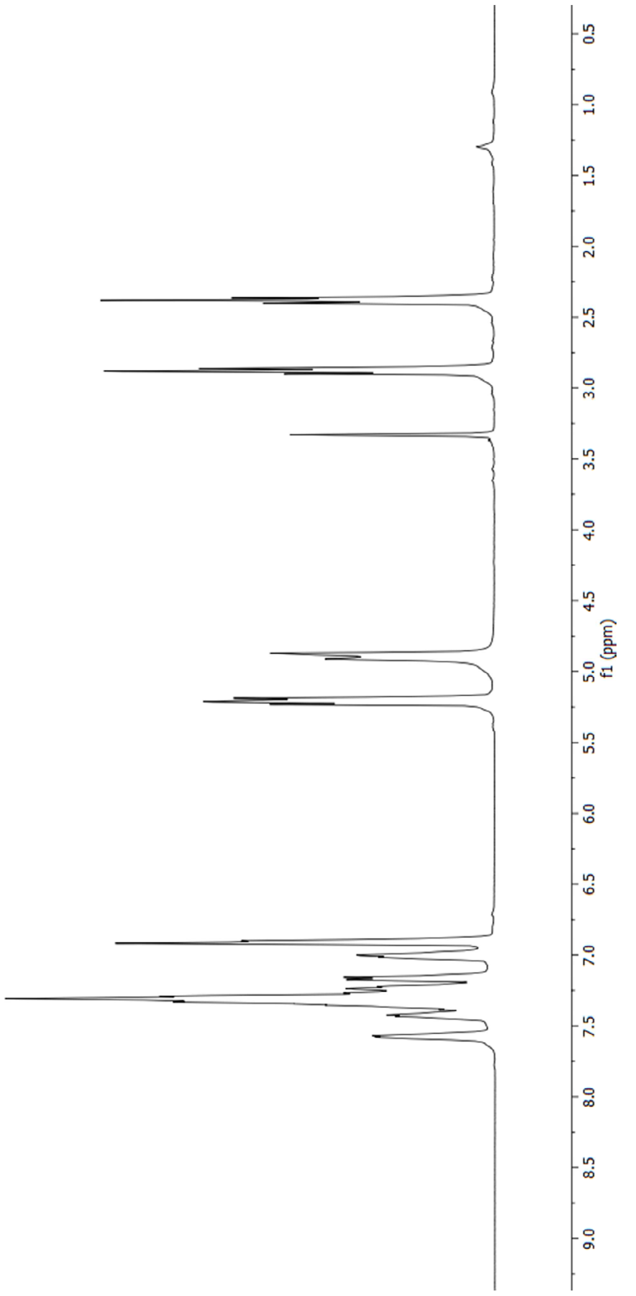

Figure S5. ^{1}H-NMR spectrum of compound **1c** (CD_3OD, 400 MHz).

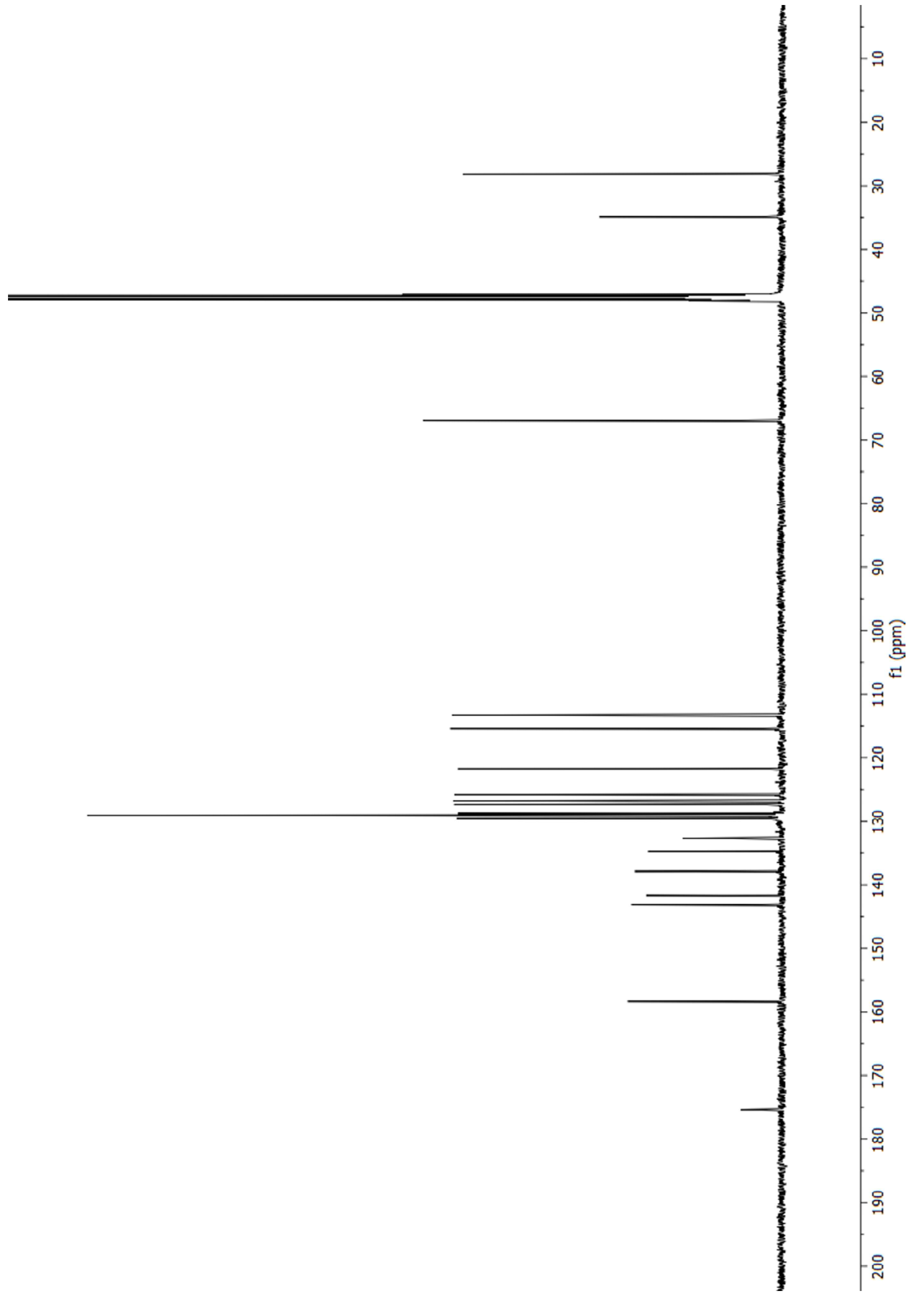

Figure S6. ^{13}C-NMR spectrum of compound **1c** (CD_3OD, 125 MHz).

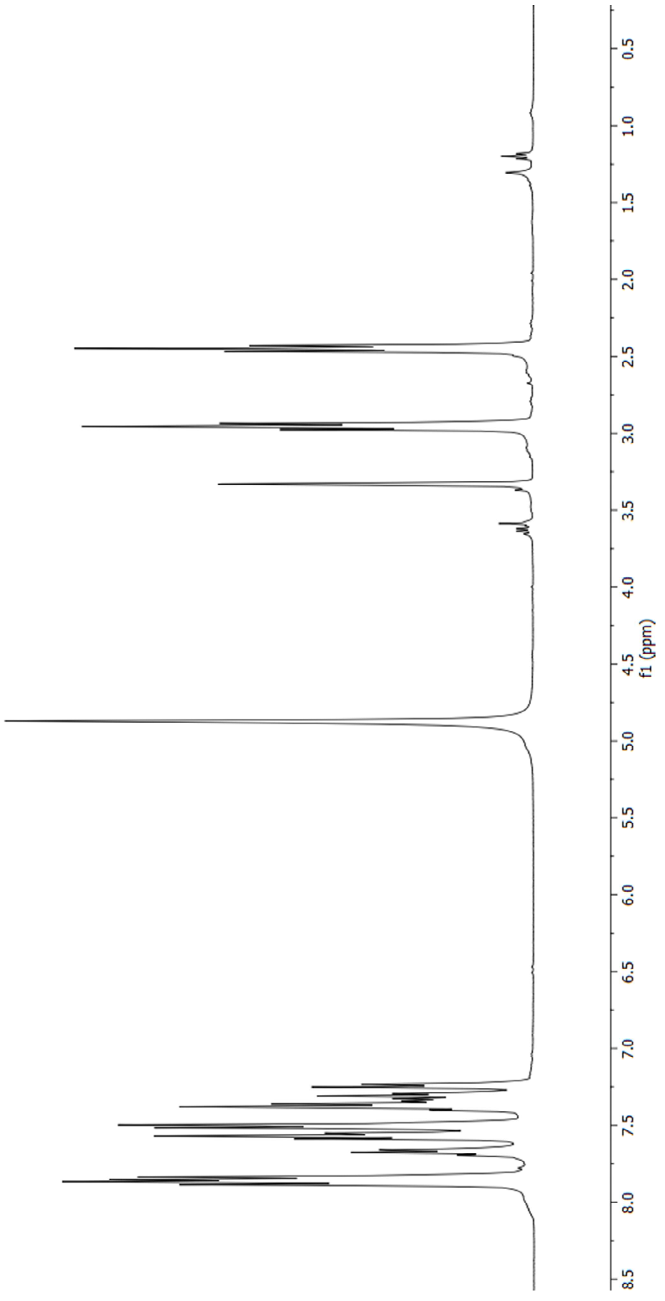

Figure S7. ^{1}H-NMR spectrum of compound **1d** (CD_3OD, 400 MHz).

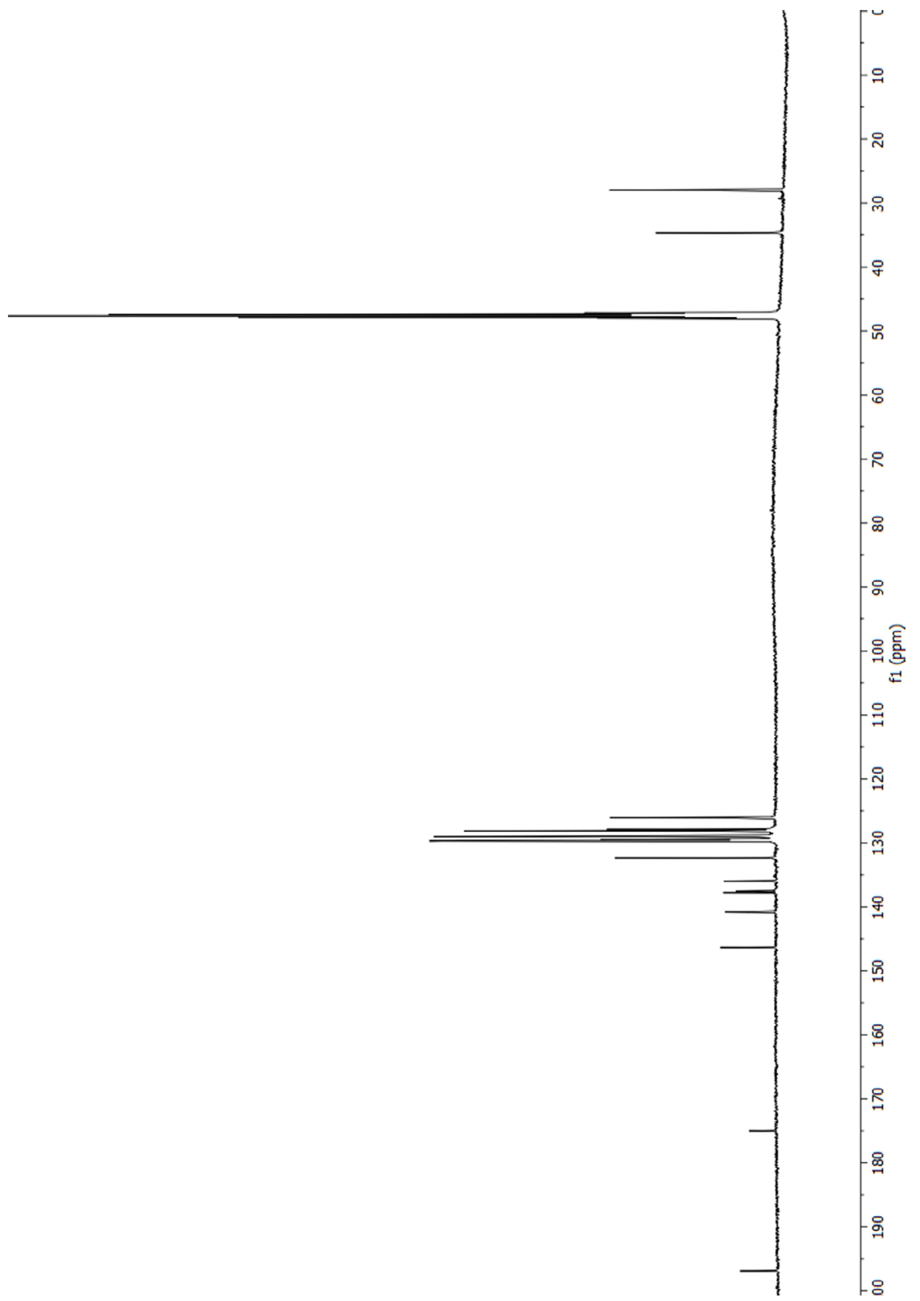

Figure S8. ^{13}C-NMR spectrum of compound **1d** (CD_3OD, 150 MHz).

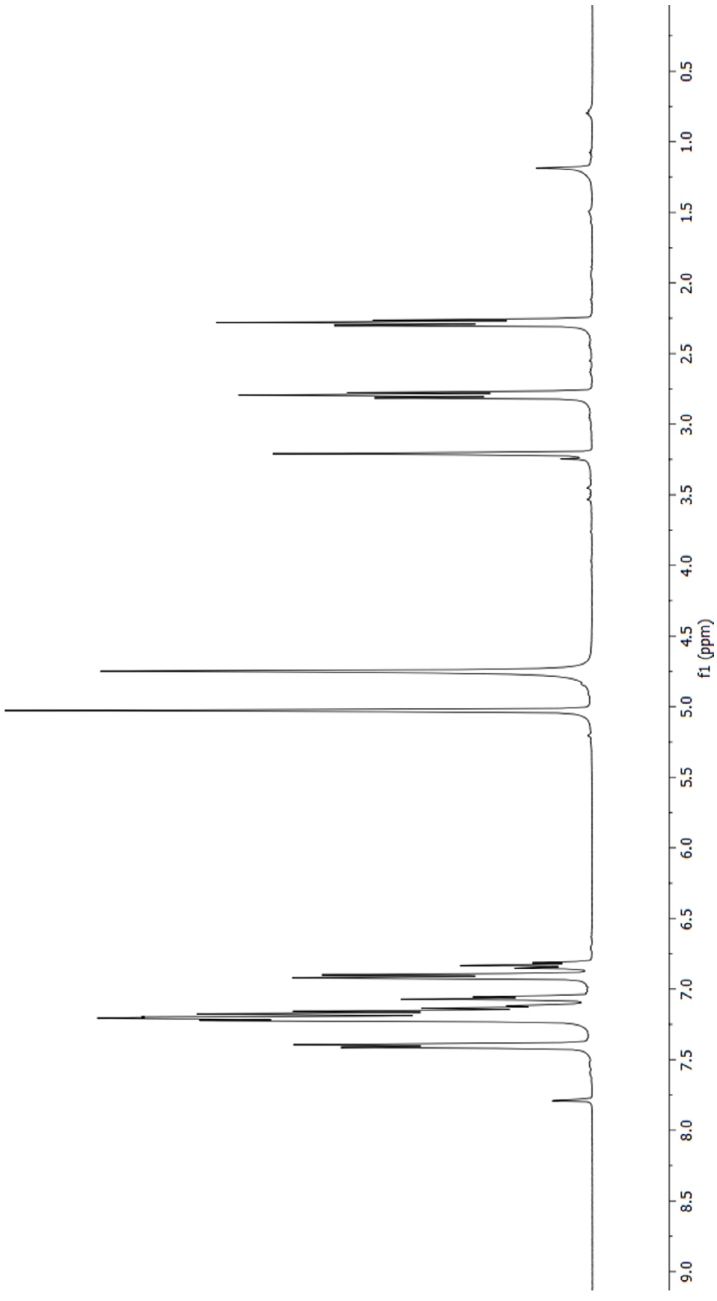

Figure S9. ^{1}H-NMR spectrum of compound **1e** (CD_3OD, 400 MHz).

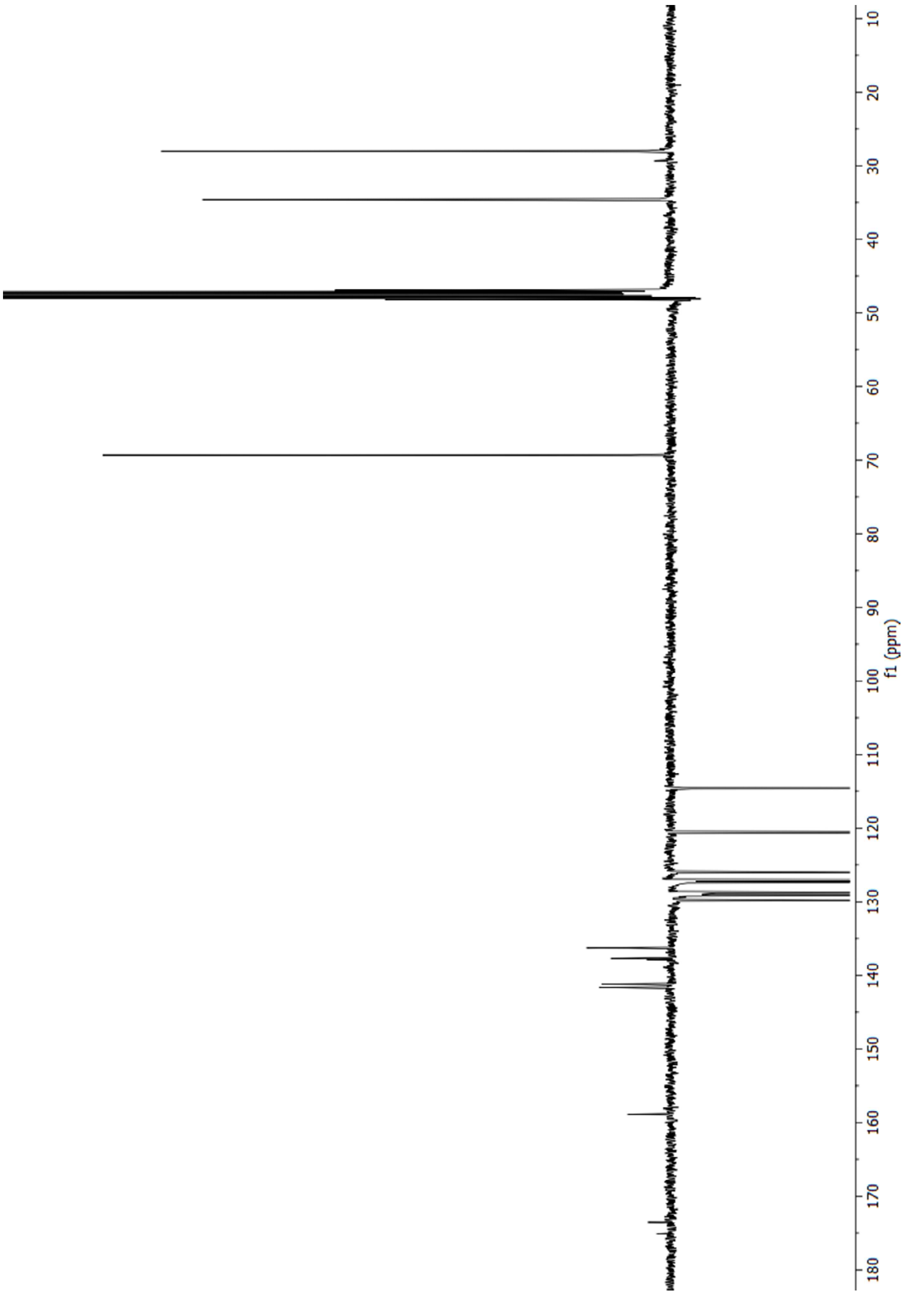

Figure S10. ^{13}C qDEPT spectrum of compound **1e** (CD_3OD, 100 MHz).

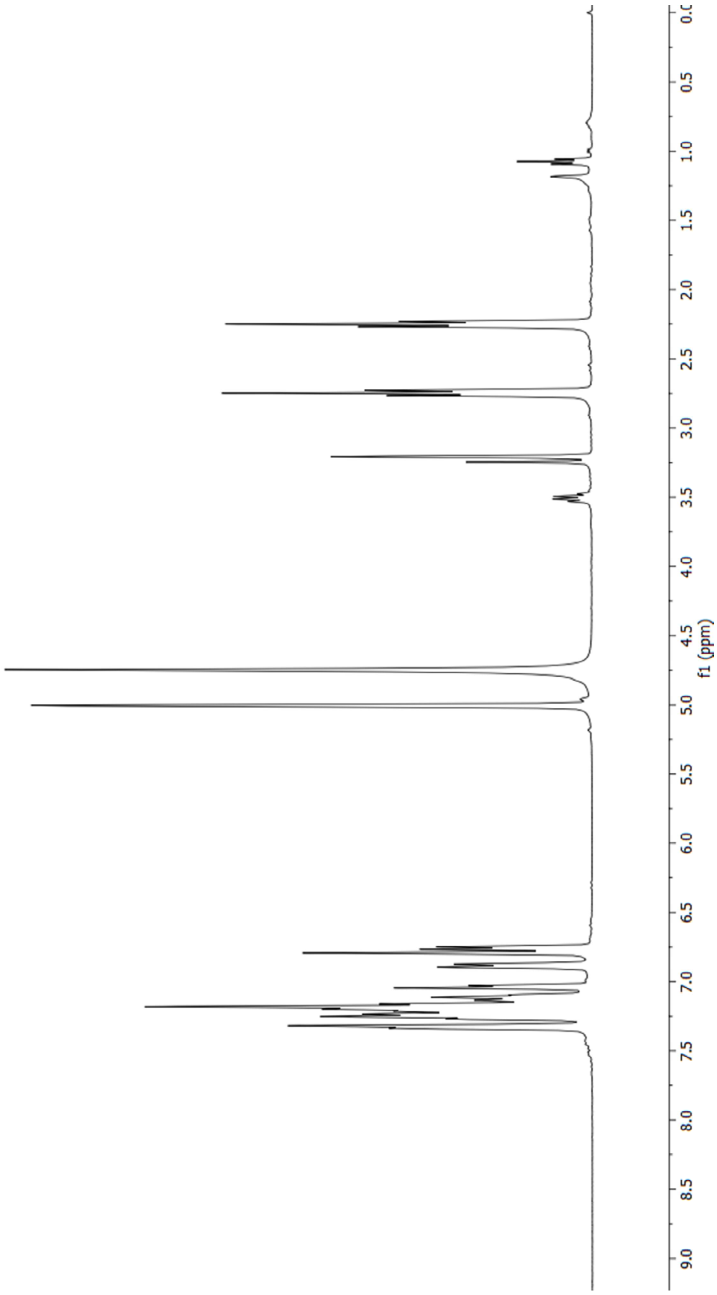

Figure S11. ^{1}H-NMR spectrum of compound **1f** (CD_3OD, 400 MHz).

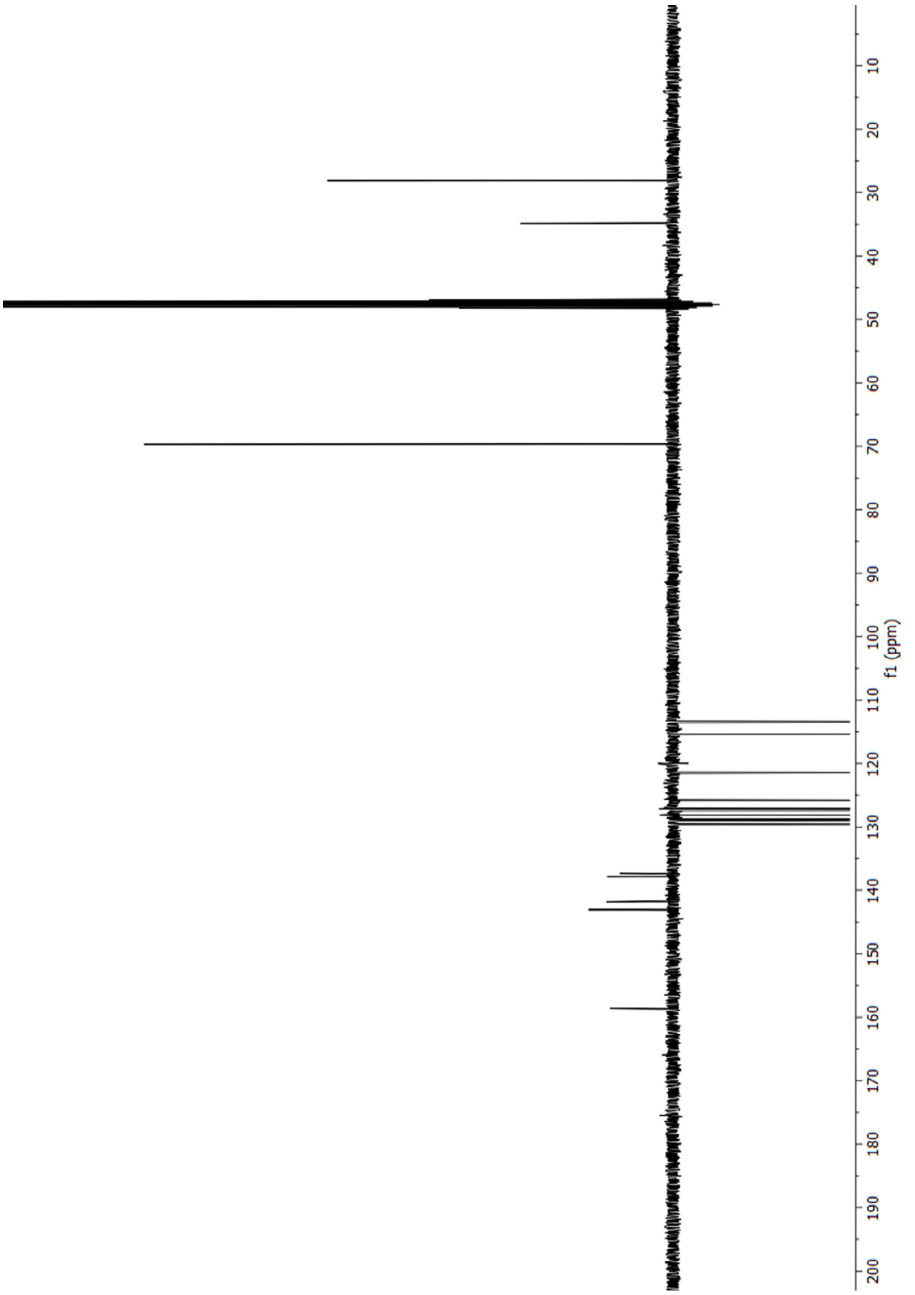

Figure S12. ^{13}C qDEPT spectrum of compound **1f** (CD_3OD, 100 MHz).

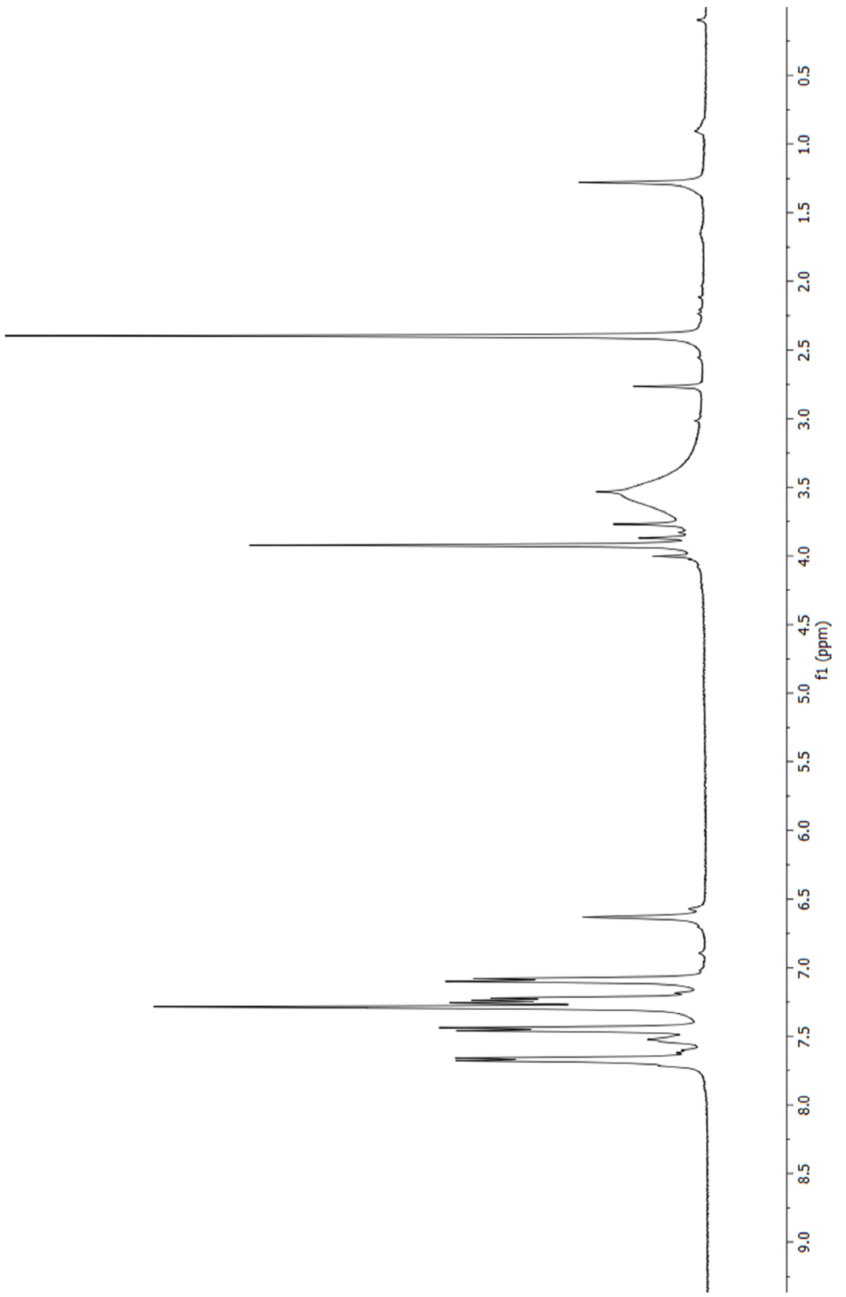

Figure S13. ^{1}H-NMR spectrum of compound **2a** ($CDCl_3$, 400 MHz).

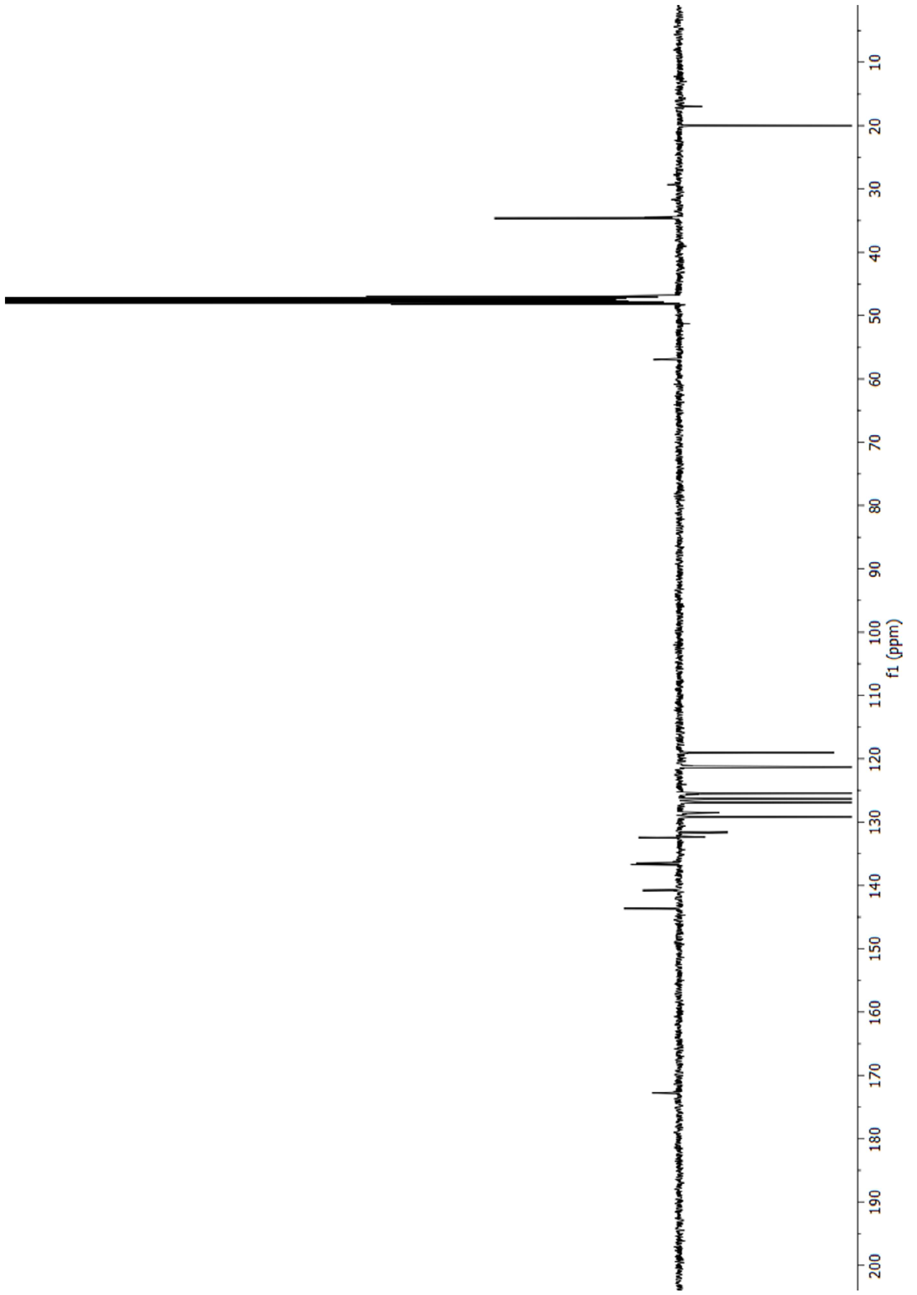

Figure S14. ^{13}C qDEPT spectrum of compound **2a** (CD_3OD, 100 MHz).

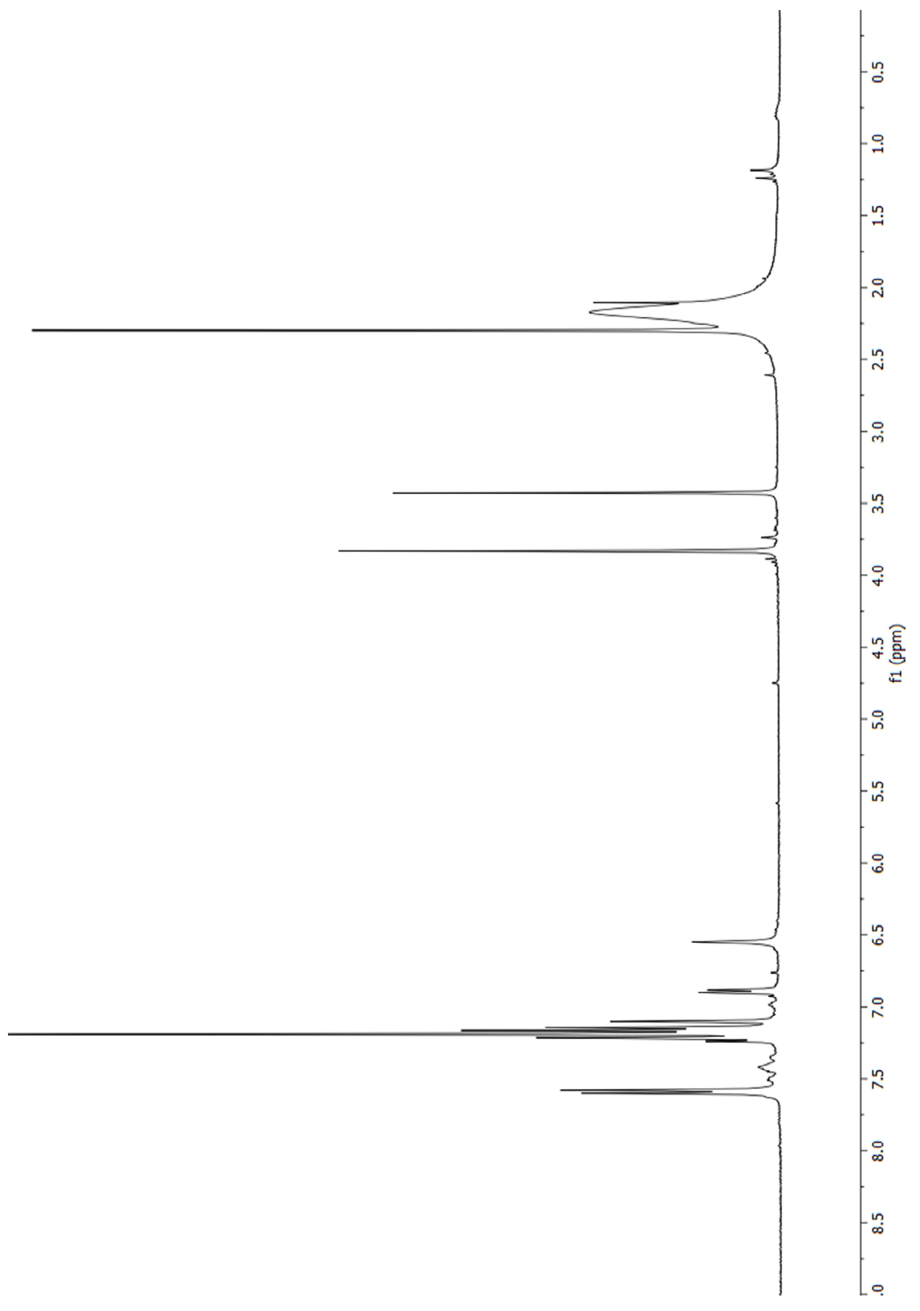

Figure S15. ^{1}H-NMR spectrum of compound **2b** ($CDCl_3$, 400 MHz).

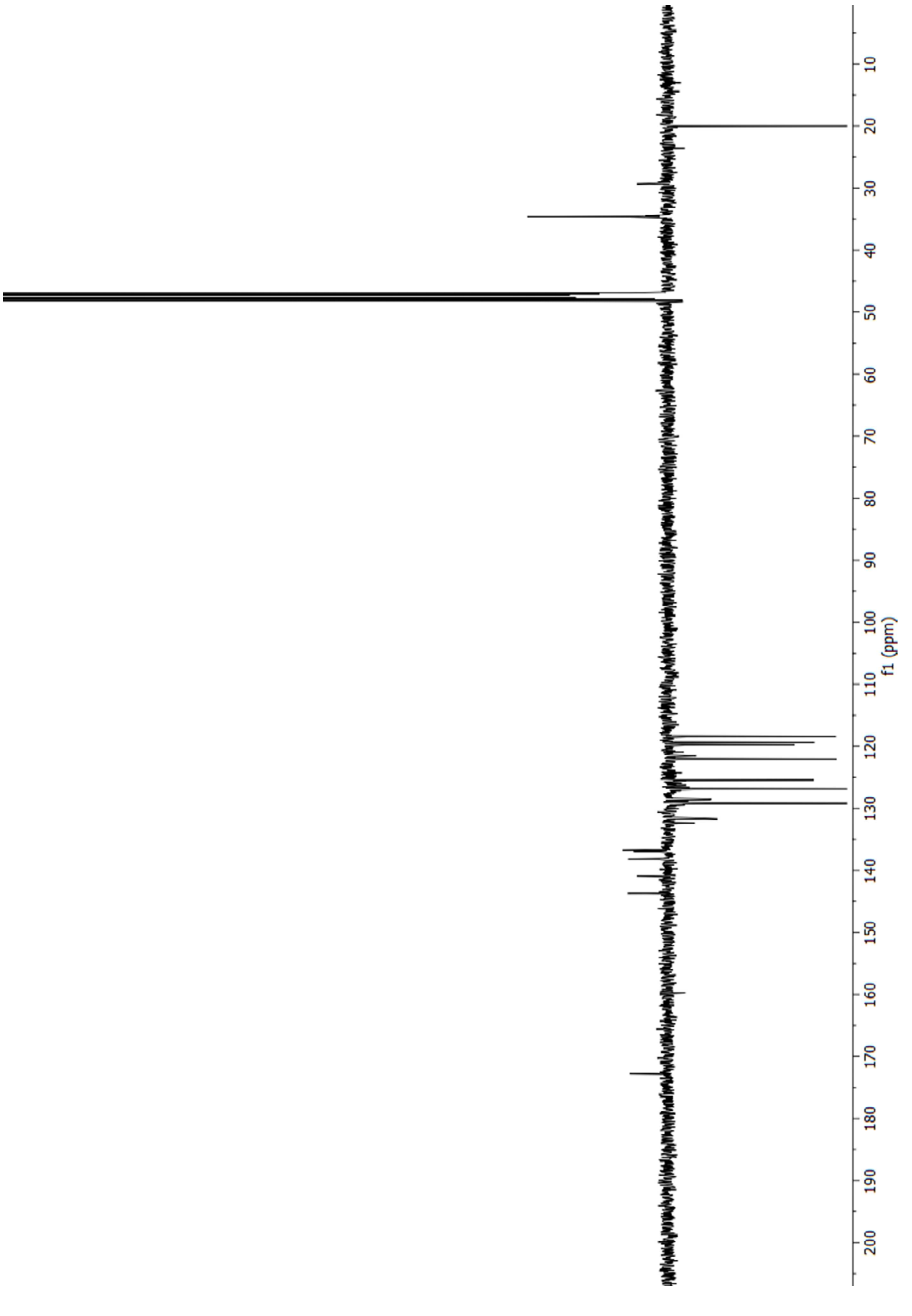

Figure S16. ^{13}C qDEPT spectrum of compound **2b** (CD_3OD, 400 MHz).

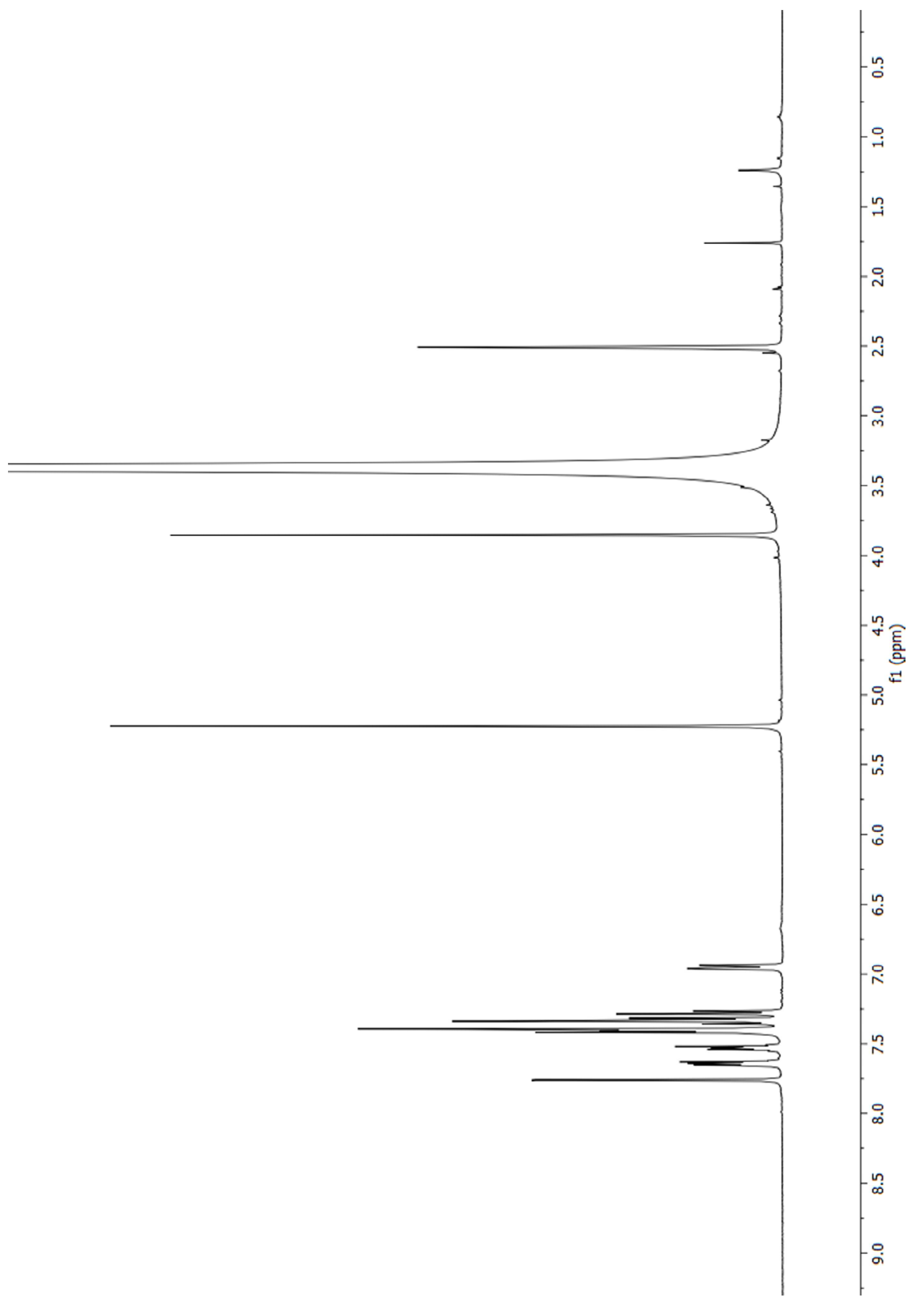

Figure S17. ^{1}H-NMR spectrum of compound **2c** (DMSO-d_6, 400 MHz).

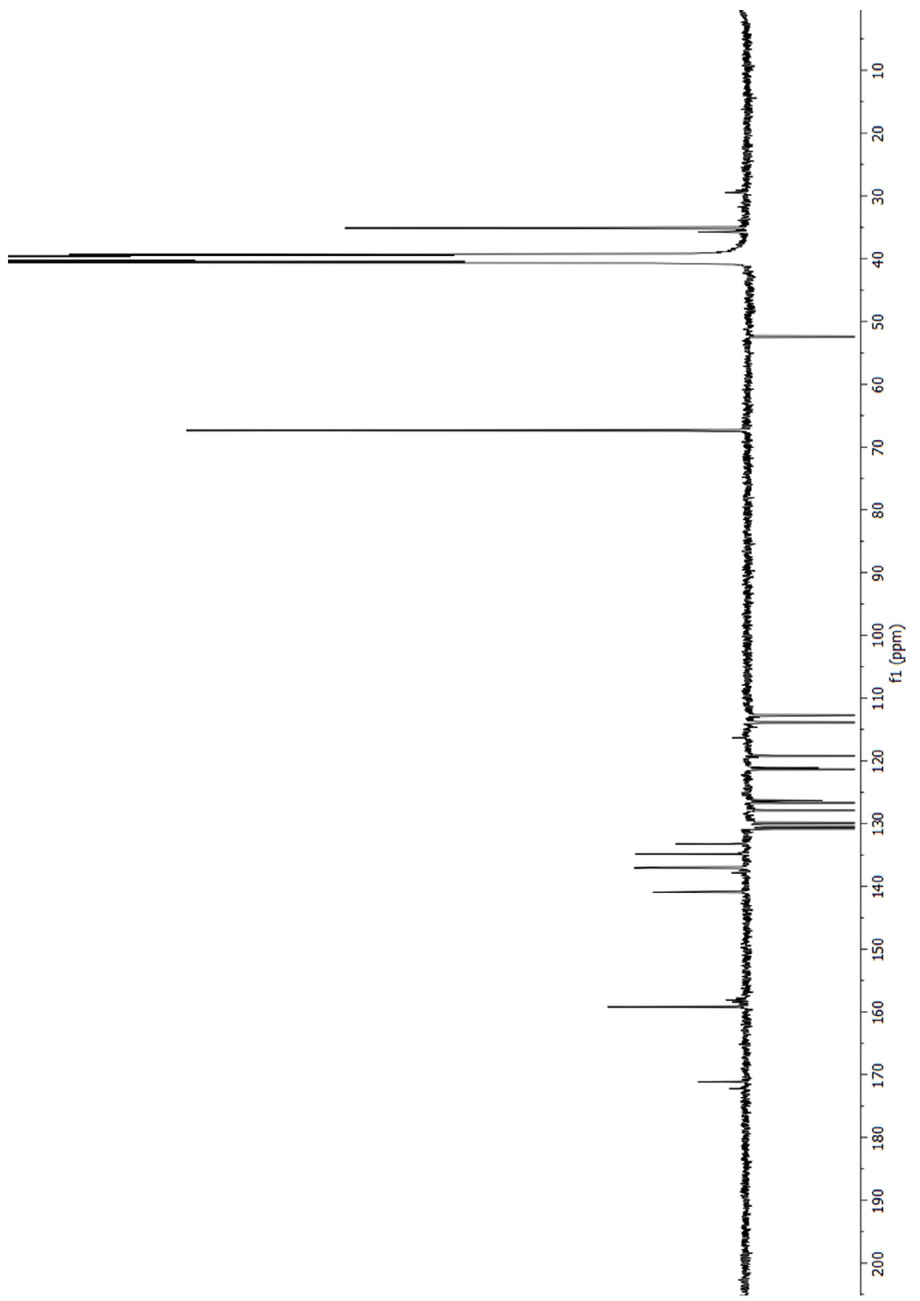

Figure S18. ^{13}C qDEPT spectrum of compound **2c** (CD_3OD, 150 MHz).

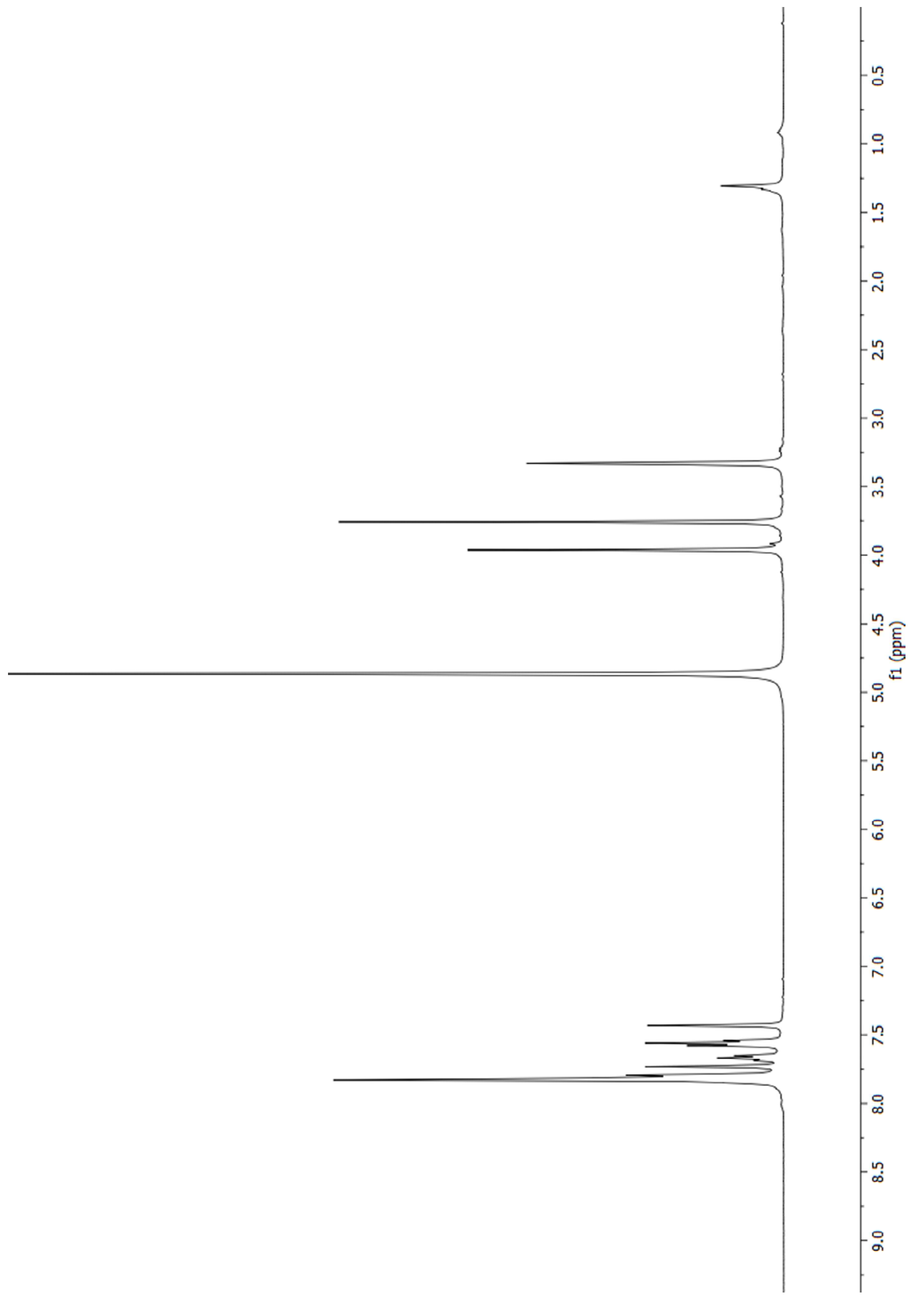

Figure S19. ^{1}H-NMR spectrum of compound **2d** (CD_3OD, 400 MHz).

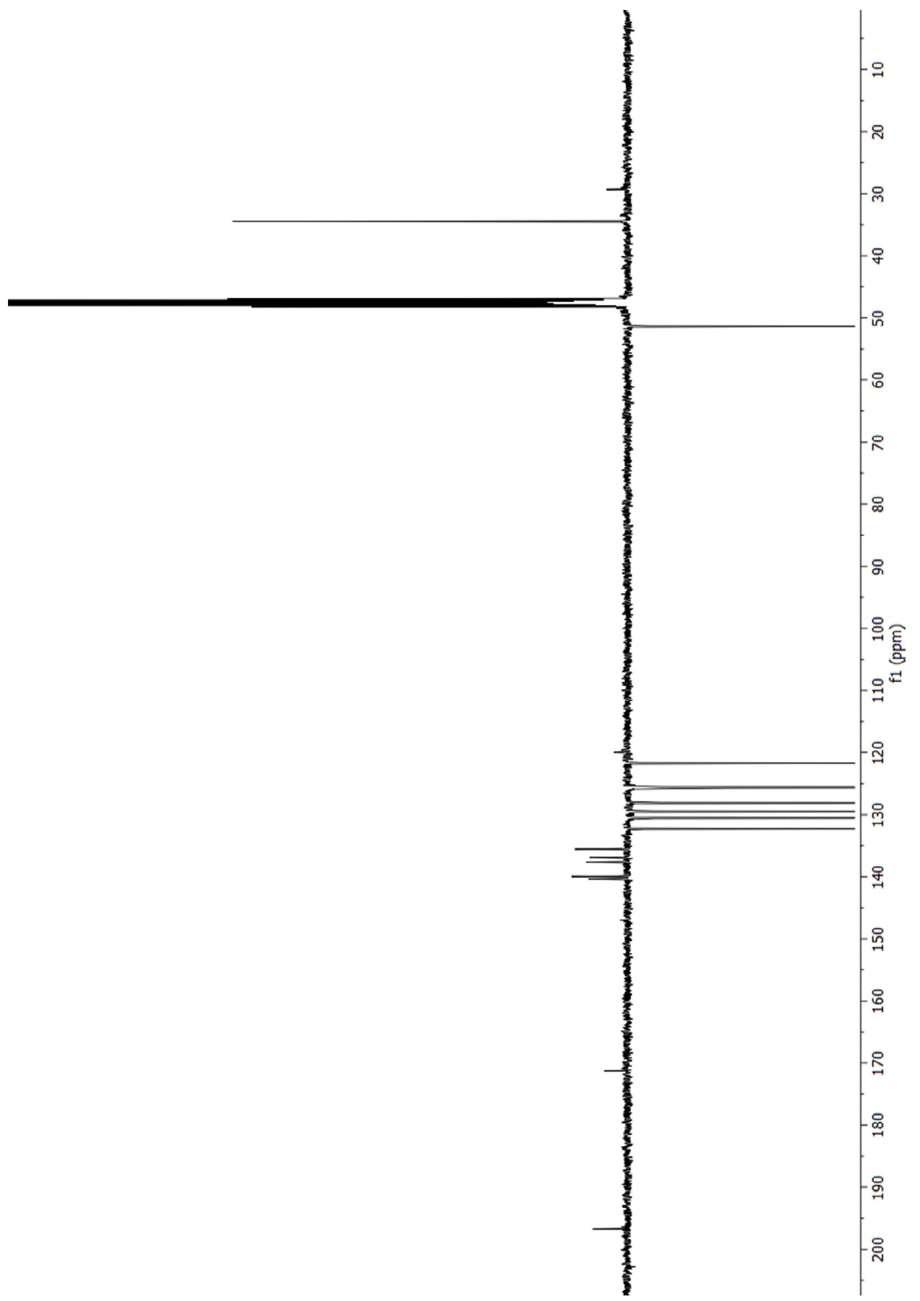

Figure S20. ^{13}C qDEPT spectrum of compound **2d** (CD_3OD, 100 MHz).

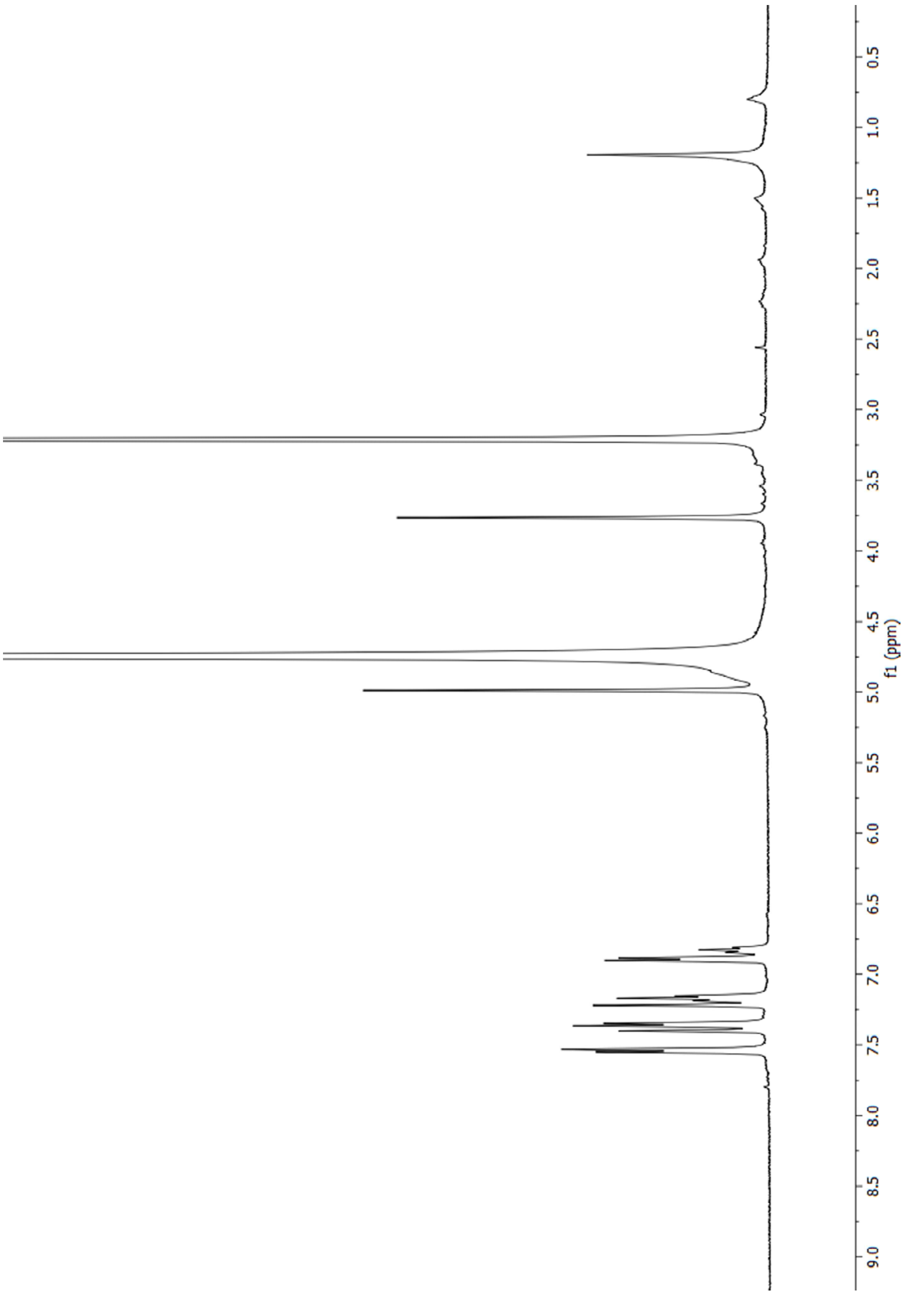

Figure S21. ^{1}H-NMR spectrum of compound **2e** (CD_3OD, 400 MHz).

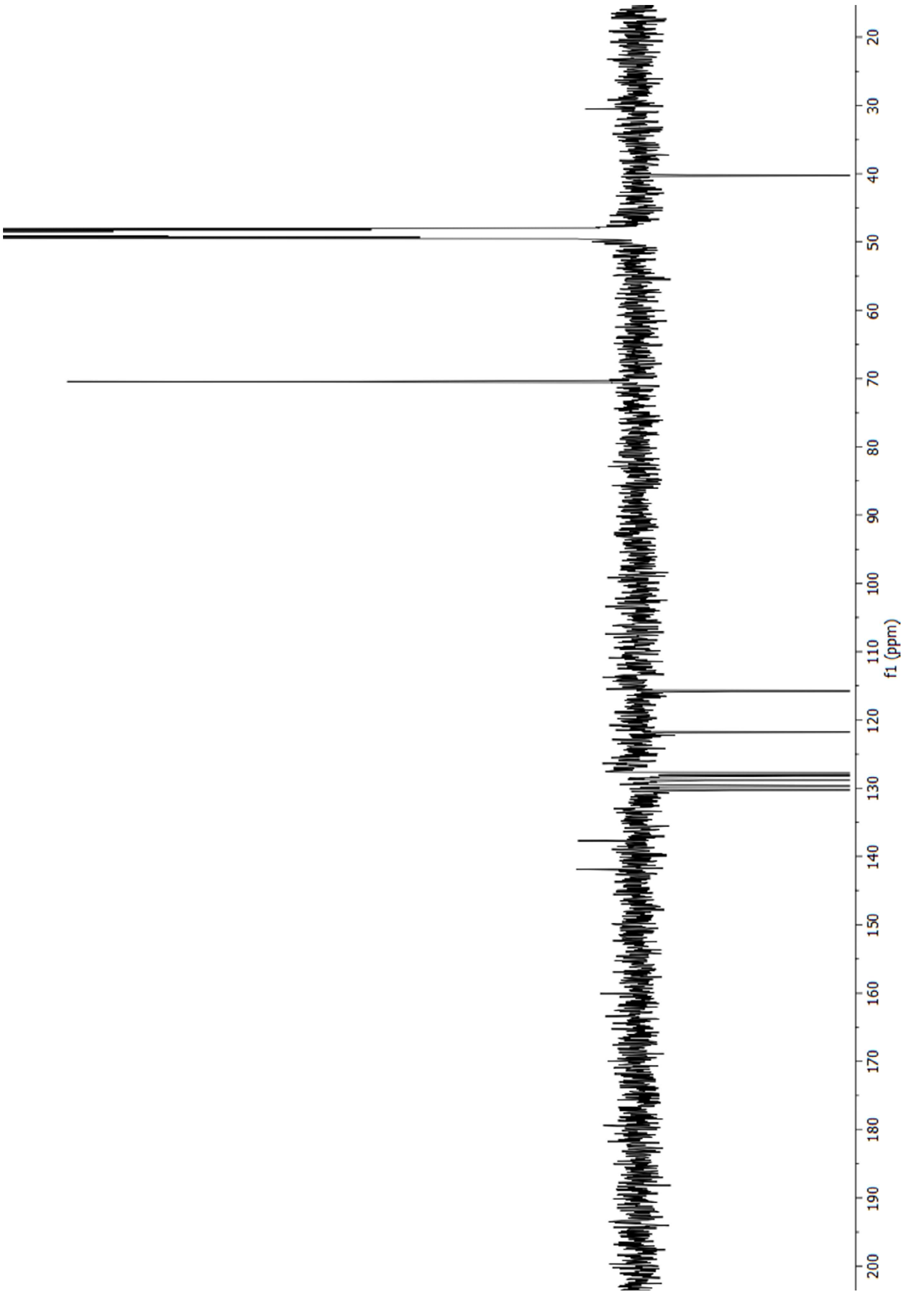

Figure S22. ^{13}C qDEPT spectrum of compound **2e** (CD_3OD, 100 MHz).

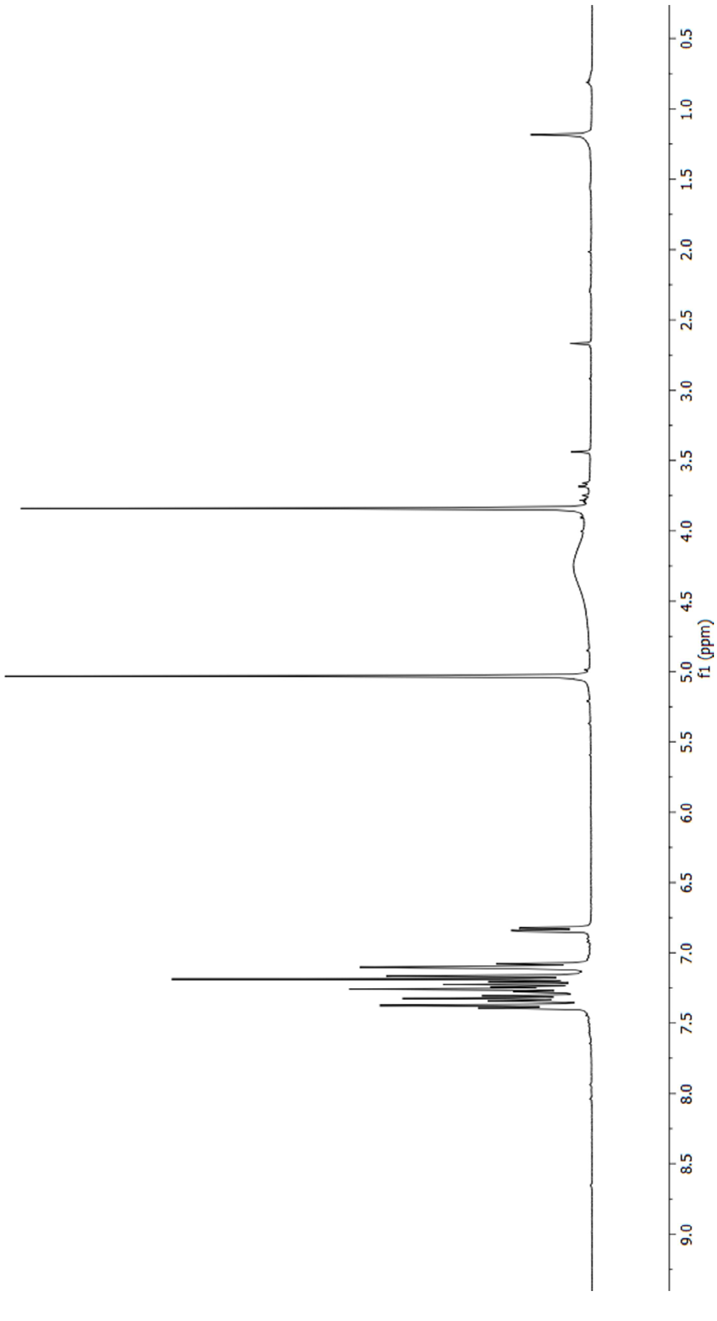

Figure S23. ^{1}H-NMR spectrum of compound **2f** ($CDCl_3$, 400 MHz).

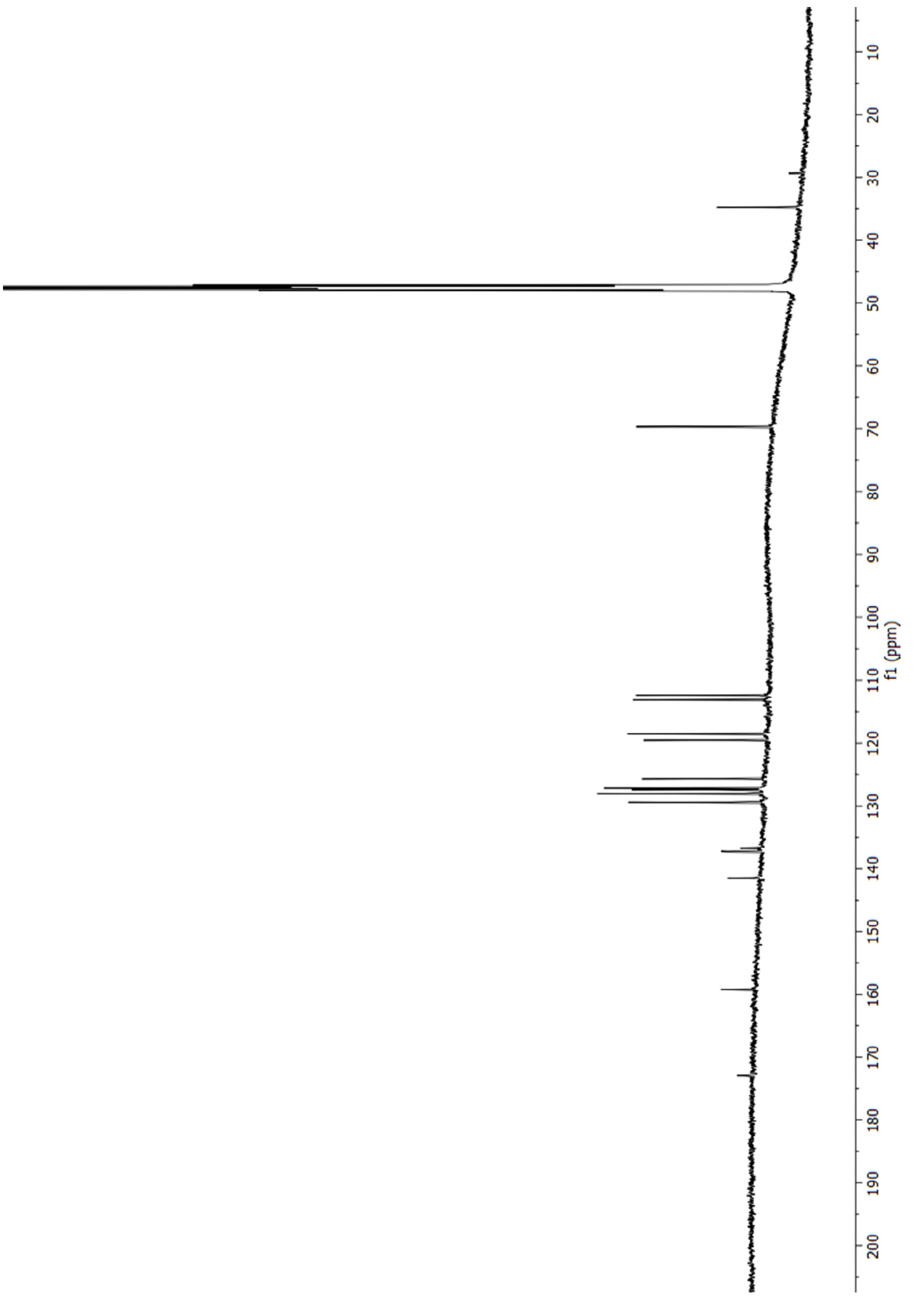

Figure S24. ^{13}C-NMR spectrum of compound **2f** (CD_3OD, 150 MHz).
